# The *bZIP53–IAA4* module inhibits adventitious root development in *Populus*

**DOI:** 10.1093/jxb/eraa096

**Published:** 2020-02-20

**Authors:** Yan Zhang, Xiaoqing Yang, Pei Cao, Zheng’ang Xiao, Chang Zhan, Meifeng Liu, Tashbek Nvsvrot, Nian Wang

**Affiliations:** 1 College of Horticulture and Forestry Sciences, Huazhong Agricultural University, Wuhan, China; 2 Hubei Engineering Technology Research Center for Forestry Information, Huazhong Agricultural University, Wuhan, China; 3 University of Antwerp, Belgium

**Keywords:** Adventitious roots, Aux/IAA genes, bZIP53, bZIP transcription factors, *IAA4*, *Populus*, salt responsive genes

## Abstract

Adventitious roots (ARs) are important for some plants that depend on clonal propagation. In this study, we demonstrate that a salt-responsive gene module is involved in the negative regulation of AR development in poplar. In this module, the expression of *bZIP53* is induced by salt stress and it encodes a transcription factor with transactivation activity. Overexpression or induced expression of *bZIP53* in poplar lines resulted in inhibition of AR growth, while heterologous overexpression of *bZIP53* in Arabidopsis resulted in a similar phenotype. Results from RNA-seq and RT-qPCR assays predicted *IAA4-1* and *IAA4-2* to be downstream genes that were regulated by bZIP53. Further investigation of protein–DNA interactions using yeast one-hybrid, electrophoretic mobility shift, dual luciferase reporter, and GUS co-expression assays also showed that *IAA4-1*/*2* were the genes that were directly regulated by bZIP53. Induced-expression IAA4-1/2 transgenic poplar lines also showed inhibited AR growth. In addition, both poplar *bZIP53* and *IAA4-1/2* showed a response to salt stress. On the basis of these results, we conclude that the bZIP53–IAA4 module is involved in the negative regulation of AR development in poplar.

## Teaser

Development of adventitious roots in poplar is negatively regulated by the *bZIP53–IAA4* module, which is responsive to salt stress.

## Introduction

Adventitious roots (ARs) are formed from non-root tissues, such as stems, leaves, and petioles. For most trees and some herbaceous species, the ability to form ARs is a major factor that determines their cultivation because these plants are mainly propagated clonally. Phytohormones, genes, physiological factors, environmental stimuli, and some chemical compounds have been reported to regulate AR development in Arabidopsis (de Klerk *et al.*, 1999; Guan *et al.*, 2015; Veloccia *et al.*, 2016). Among these, the phytohormone auxin plays central roles in regulating all of the processes involved in AR development (Della Rovere *et al.*, 2013; Pacurar *et al.*, 2014). Other phytohormones, including cytokinins, abscisic acid (ABA), jasmonic acid (JA), and ethylene (ET), also positively or negatively regulate AR development at some or all the stages of plant growth and development (Gutierrez *et al.*, 2012; Isner *et al.*, 2012; Li *et al.*, 2014; Mauriat *et al.*, 2014; Veloccia *et al.*, 2016; Mao *et al.*, 2019). Overall, AR development in plants is an extremely complex process, and auxin or auxin-related genes play a major role as regulators.

Basic region/leucine zipper motif (bZIP) proteins are some of the most important transcription factors (TFs) in plants. bZIP TFs regulate many biological processes, including organ development, stress defence, nutrient assimilation, and seed maturation (Jakoby *et al.*, 2002; Alves *et al.*, 2013; Ali *et al.*, 2016). Genome-wide identification of bZIP TFs has been performed in a number of species and they are known to be 75 distinct members in Arabidopsis, 170 in maize, 77 in cassava, 121 in banana, and 191 in wheat (Jakoby *et al.*, 2002; Wei *et al.*, 2012; Hu et al., 2016*a*, 2016*b*; Agarwal *et al.*, 2019). Some bZIP TFs show binding activity to the G-box (core sequence ACGT) (Foster *et al.*, 1994; Izawa *et al.*, 1993; Sibéril *et al.*, 2001), while others can bind with TGA (core sequence TGACG) or AC (core sequence ACTCAT) elements (Berendzen *et al.*, 2012). In Arabidopsis, bZIP TFs are classified into 11 groups (Jakoby *et al.*, 2002). Many members in the S-group respond to abiotic stresses, including low energy and salinity. The core S-group members, bZIP53 and bZIP1, are activated by starvation and reprogram the downstream primary metabolism (Weltmeier *et al.*, 2006; Dietrich *et al.*, 2011). Another member, bZIP11, activates auxin-mediated transcription by recruiting the histone acetylation machinery (Weiste and Dröge-Laser, 2014), and it also links low-energy stress with auxin-mediated control of root growth (Weiste *et al.*, 2017). bZIP11 promotes the expression of *IAA3* and results in negative regulation of root elongation. In poplar, a bZIP1-like protein enhances lateral root formation and biomass growth under drought stress (Dash *et al.*, 2017), while in tobacco the bZIP TF BZI-1 binds the *Gretchen Hagen3* (*GH3*) promoter *in vivo* and modulates auxin-induced transcription (Heinekamp *et al.*, 2004). These findings suggest that members of the bZIP S-group might be involved in both auxin and abiotic stress regulation. Since auxin has central roles in AR development, some members in the this group also positively or negatively regulate AR development upon abiotic stress.

Salt stress is one of most common abiotic stress factors in plant growth (Zhu, 2003), and upon exposure to it plants usually deploy responses at the molecular, cellular, and physiological levels in order to overcome the unfavourable growth conditions (Chinnusamy *et al.*, 2006; Zhang and Shi, 2013; Deinlein *et al.*, 2014). Salt stress will usually result in growth inhibition (Golldack *et al.*, 2014), and AR development can also be affected. Generally, a high level of salt stress can severely inhibit AR development, while low levels can promote AR formation in some cases. Plants usually reprogram their gene/protein expression as a response to stress conditions (Krasensky and Jonak, 2012; Golldack *et al.*, 2014). Although there have been a lot of studies focusing on gene regulation under salt stress, salt-induced growth inhibition has not been comprehensively investigated, especially for AR development. Uncovering the molecular mechanisms involved in salt-inhibited AR growth will help in future strategies for the development of salt-resistant plants.

Poplars, which are fast-growing and highly adaptable, have been considered as one of the most important tree species in the worldwide forestry industry. Commercially, poplars are mainly propagated by cuttings, and there is a great amount of variation in the ability to form ARs among different species. Although the mechanisms involved in AR formation and elongation have been extensively studied in the model species Arabidopsis, large differences exist between the two plants. ARs are usually induced at the hypocotyl in Arabidopsis (Welander *et al.*, 2014), while cuttings are used for AR induction in poplar. In addition, some poplars show pre-formed primordia before the cuttings are prepared (Zhang *et al.*, 2019). These visible differences suggest that the mechanisms involved in AR development between poplar and Arabidopsis are not completely be the same. As a model tree species, there have been several studies focusing on AR development in poplar, and a number of genes have been shown to be involved in its regulation (Ramírez-Carvajal *et al.*, 2009; Rigal *et al.*, 2012; Trupiano *et al.*, 2013; Wuddineh *et al.*, 2015; Xu *et al.*, 2015; Yordanov *et al.*, 2017; Li *et al.*, 2018; Liu *et al.*, 2019), including large-scale data analyses identifying regulators (Ribeiro *et al.*, 2016; Zhang *et al.*, 2019) and pharmacological assays of physiological regulators (Gou *et al.*, 2010; Zhang *et al.*, 2019). However, precise knowledge of this important biological process, especially the link between the stress response and AR development, remains elusive.

In this study, we identified the poplar TF bZIP53, a core member of the S-group, and its gene showed a response to salt treatment. Overexpression and induced-expression lines of poplar *bZIP53* were generated both in poplar and Arabidopsis, and AR development in the transgenic plants was repressed. A transcriptomic analysis identified *IAA4-1* and *IAA4-2* (homologous to *IAA3/4* of Arabidopsis) as candidates for downstream regulation by poplar bZIP53. Using protein–DNA interaction assays, we found that that bZIP53 directly regulated *IAA4-1/2* in poplar. Induced-expression lines for poplar *IAA4-1/2* were generated, and these plants also showed repressed AR development. On the basis of these results, we conclude that poplar bZIP53 is salt-responsive and negatively regulates AR development by directly binding to *IAA4-1* and *IAA4-2*.

## Materials and methods

### Plant materials, growth conditions, and treatments

Two *Populus* lines, ‘NL895’ and ‘Shanxinyang’, were used in this study. ‘NL895’ is a hybrid variety generated through the cross between *Populus deltoides* and *P. euramericana*, while ‘Shanxinyang’ is a hybrid variety generated through the cross between *P. davidiana* and *P. bolleana*. Plants were generated by tissue culture conducted at 25–28 °C with a 16/8-h photoperiod according to the protocol described by Wang *et al.* (2011). Arabidopsis was grown in soil in a growth chamber at 19–22 °C with 7500 lux light intensity, and a 12/12-h photoperiod.

To examine the expression of selected poplar genes in response to salt and ABA treatments, 1-month-old tissue-culture plantlets of ‘NL895’ with uniform growth (~10 cm in length) were acclimatized and propagated in a hydroponic propagation system for 10–15 d under normal growth conditions (25 °C with a 16/8-h photoperiod and 7500 lux light intensity). The hydroponic propagation system was similar to the one described by Zhang *et al.* (2019). Plants that reached ~15 cm with uniform growth were selected, and 150 mM NaCl or 100 μM ABA was applied via the hydroponic solution. Roots and stems that were in the solution were collected for subsequent RNA isolation. Control samples were taken at 0 h, and treatment samples were taken between 1–24 h. At each time point, at least 15 plants were sampled, and five were pooled to form each biological replicate. To examine the expression of poplar *bZIP53* in different tissues, tissue-cultured plantlets of ‘NL895’ at 20 d old with uniform growth were transplanted into 1-l pots containing soil and grown in a chamber with regular irrigation for 1 month under 25 °C with a 16/8-h photoperiod and 7500 lux light intensity. Samples of roots, young stems, shoots with young leaves, mature leaves, and petioles were collected for subsequent RNA isolation. Tissues from at least five plants were pooled to form each biological replicate, and three replicates were used.

### RNA isolation and reverse-transcription quantitative PCR analysis

The procedures for RNA isolation, quality examination, cDNA synthesis, and RT-qPCR assays were identical to those of our previous study (Zhang *et al.*, 2019). Two reference genes, *ACTIN* and *UBQL* (Supplementary Table S1 at *JXB* online), were used to standardize relative expression values, and the 2^–ΔΔ*C*T^ method was used to calculate the relative gene expression based on the RT-qPCR data (Livak and Schmittgen, 2001).

### Generation of transgenic Arabidopsis and poplar

The modified binary vector 2301S, which harbours a 2×35S promoter before multiple cloning sites and a *35S::GUS*, was used to generate the overexpression (OE) construct. The binary plasmid PER8, which harbours a XVE inducible system before multiple cloning sites, was used for construction of the induced-expression (IE) construct (Zuo *et al.*, 2000). The coding sequences (CDSs) of poplar *bZIP53*, *IAA4-1*, and *IAA4-2* were amplified using cDNA prepared from salt-treated roots and stems of ‘NL895’. The primers for these PCR amplifications were listed in Supplementary Table S1. The CDSs were cloned into the plasmids 2301S and PER8, generating the vectors OE-bZIP53, IE-bZIP53, IE-IAA4-1, and IE-IAA4-2. Schematic diagrams for the constructs of OE-bZIP53 and IE-bZIP53 are illustrated in Supplementary Figs S3a and S6a, respectively.

The Columbia ecotype of Arabidopsis (Col-0) was transformed using *Agrobacterium tumefaciens* GV3101 possessing the OE-bZIP53 or *35S::bZIP53:GFP* construct. T_3_ lines with a single transgenic copy were screened, and their seeds were harvested for subsequent phenotypic assays. For the production of transgenic poplar material, ‘NL895” was used to generate OE lines and ‘Shanxinyang’ was used to generate IE lines. The procedures used for both poplar varieties were similar, and the procedures are detailed in Wang *et al.* (2011). For confirmation of incorporation of DNA, forward or reverse primers were designed according to the sequences from 2301S or PER8 together with reverse or forward primers from the CDSs of *bZIP53 IAA4-1*, or *IAA4-2*. For confirmation of RNA levels, RT-qPCR was used to verify that *bZIP53*, *IAA4-1*, and *IAA4-2* were constitutively or inductively expressed. Since *35S::GUS* was incorporated into the 2301S binary plasmid, GUS staining was applied for screening of protein levels. GUS staining was performed according to Lee and Schoffl (1995).

### Subcellular localization assays

The CDS of *bZIP53* (without a stop codon) was amplified and cloned into pLGFP1301 to create the *35S*::*bZIP53::GFP* construct, which was transformed into *A. tumefaciens* GV3101. Leaves from 5- to 6-week-old *Nicotiana benthamiana* were used for the transient assay by *Agrobacterium*-mediated transfection. After 2–3 d of culture, the transient transformed leaves were observed using a fluorescence microscope (Leica, DM2500). This construct was also used to generate stable transgenic lines of Arabidopsis.

### Transactivation activity assays

The CDS and different fragments of poplar *bZIP53* were separately cloned into the PGBKT7 plasmid, allowing them to fuse with the GAL4 binding domain. The constructs were transformed into the yeast strain AH109, and successfully transformed clones were selected on SD/–Trp medium. SD/–Trp-His–Ade+X-α-Gal medium was used to test the transactivation activity for the CDS and the different fragments of *bZIP53* according to both the growth status and the blue colour reaction of the different transformed yeast strains.

### Phenotypic assays of AR development for transgenic Arabidopsis and poplar

Homozygous T_3_ transgenic lines of Arabidopsis were used to observe AR development following the procedure described by Sorin *et al.* (2005). Seeds of Arabidopsis were germinated in the dark at 22 °C on plates of MS medium. After 5 d, the plates were transferred to a photoperiod of 12/12 h at 7500 lux and 22 °C day/night temperature. ARs were scored for 10 d after the plates were transferred to the light. At least 50 seedlings were scored in each plate, and three plates were used for each transgenic and wild-type (WT) line.

To examine the AR development of transgenic poplar lines in woody plant medium (WPM), 1-month-old tissue-cultured plantlets were prepared for the transgenic and WT lines. Only plants with uniform growth were used for the phenotypic assays, and microcuttings with 2–3 upper leaves and an apical bud (~2–3 cm) were excised. For the OE poplar lines, the excised microcuttings were inserted into WPM, while for IE poplar lines they were inserted into WPM supplemented with 10 µM oestradiol. The number of ARs was observed every day, together with their fresh weight and length. Similarly, AR development of transgenic poplar lines was examined for plants growing in soil. Microcuttings were inserted into soil (sand:peat, 70:30, v/v) and grown in a chamber under a 16/8-h photoperiod (7500 lux at 25 °C, and irrigated with 50× Hoagland’s solution every 2–3 d. For the oestradiol treatment, 1 ml of 10 µM solution was added to the stem base of each microcutting every 2–3 d.

To examine the effects of exogenous application of auxin, microcuttings of the WT and IE lines were propagated for 8 d in WPM with either supplementation of 10 µM oestradiol and 1.0 mg l^–1^ IAA or with only supplementation of 10 µM oestradiol. The preparation of the initial microcuttings and growth conditions were identical to the experiments for AR development observation in WPM described above

### Yeast one‐hybrid assays

The 1.6-kb promoter sequence (designated as IAA4-Pro) of poplar *IAA4-1* (*Potri.005G218200*) was amplified by PCR with the primer pairs IAA4-1-Pro-1F/R (Supplementary Table S1). The 340-bp sequence including the G-Box motif in IAA4-Pro was PCR-amplified using the primer pairs Gbox-F/R, and this fragment was designated as G-BoxS. The mutated G-BoxS (mG-BoxS) showed an identical sequence with G-BoxS, except that the 6-bp G-Box was synthesized. The whole promoters of IAA4-Pro, G-boxes, and mG-BoxS were cloned into the plasmid pAbAi vector to create three bait constructs. The CDS of poplar *bZIP53* was cloned into the plasmid pGADT7 to create the prey construct pGADT7-bZIP53. Yeast one-hybrid (Y1H) assays were performed using the Matchmaker® Gold Yeast One-Hybrid Library Screening System (Clontech) according to the manufacturer’s instructions.

### Dual-luciferase reporter and GUS assays

The CDS of *bZIP53* was cloned into the vector pGreenII 62-SK, and this construct was used as an effector plasmid. The *IAA4-1* promoter IAA4-Pro was cloned into the vector pGreen II 0800-LUC to obtain the reporter plasmids. The empty vector of pGreenII 62-SK was used as a control effector, while the introduced bZIP53 vector of pGreenII 62-SK was used as a treatment effector. *Agrobacterium tumefaciens* GV3101 processing the pSoup-P19 plasmid was transformed with the plasmids for the effectors and reporters, and then leaves from 5- to 6-week-old *N. benthamiana* plants were co-transformed with the strains using a needleless syringe. After 2–3 d of culture in the dark, a Dual-Luciferase Reporter Assay System (Promega, E1910) was used to qualify LUC and REN activity according to the manufacturer’s instructions. At least six biological replications were conducted for each co-transformation, and the ratios of LUC to REN for both treatments and controls were calculated to examine the binding activity of bZIP53 to IAA4-Pro. All of the procedures were performed according to Hellens *et al.* (2005).

For the GUS transient overexpression assays, the CDS of *bZIP53* was cloned into the vector pGreenII 62-SK, and this construct was used as an effector plasmid. The promoter IAA4-Pro, G-boxS, and mG-boxS were cloned into the vector DX2181 to obtain three reporter plasmids. The co-expression transformation of the effector and reporter plasmids was identical to the procedure used for the dual-luciferase reporter assays. GUS staining was performed according to Lee and Schoffl (1995).

### Electrophoretic mobility shift assays

The *bZIP53* CDS (without a stop codon) was cloned into the plasmid pHMGWA, generating a 6×His::MBG::bZIP53::6×His construct. This construct was expressed in *Escherichia coli* Rosetta (DE3), and the fusion protein was purified with a Ni Sepharose 6 Fast Flow Kit (GE Healthcare). A 45-bp sequence processed with a G-Box in the promoter of poplar *IAA4-1* (aaattagaggtcccacattc*acgt*gggaccctcagttcacatggg; core bases of the G-Box are in italics), designated as a Probe, was synthesized and labelled with biotin at the the 3′-hydroxyl end of the sense strand; in addition, the same 45-bp sequence with ‘caaaag’ substituted for the G-Box was designated as mProbe and was also synthesized and labelled with biotin at the 3′-hydroxyl end of the sense strand. An unlabelled Probe was also synthesized and used as the competitor. Different mixtures of 6×His::MBG::bZIP53::6×His protein and Probe/mProbe were employed for electrophoresis on a 6.0% polyacrylamide gel and then transferred to a nylon membrane. The membrane was passed through a CCD imaging device (Molecular Imager ChemiDoc XRS+) for moderate exposure and imaging.

### Statistical and bioinformatic analyses

Statistical analyses were performed using the R software (https://www.r-project.org/). Student’s *t*-tests and one‐way ANOVA followed by Duncan’s multiple comparisons were employed for differential comparisons of two and more samples, respectively. The procedure for transcriptomic analysis based on RNA-seq was performed similarly to our previous study (Zhang *et al.*, 2019). Briefly, high-quality RNA of the poplar WT and transgenic IE-bZIP53 lines was isolated and sequenced using a Hiseq 10× platform. Clean reads were mapped onto the *P. trichocarpa* genome version 3.0 (https://phytozome.jgi.doe.gov/) using TopHat version 2 software (Ghosh and Chan, 2016). The read count for each sample was calculated using ‘htseq-count’ with the ‘unique mapping’ parameter (Anders *et al.*, 2015), and only the genes with an average read count greater than 2 in at least one sample were kept for further analysis. Differentially expressed genes were identified using the EdgeR software with a false discovery rate less than 0.05 and a fold-change greater than 2.0 (Robinson *et al.*, 2010).

## Results

### Poplar bZIP53 is a transcription factor, and its expression is induced by salt stress

In a previous study, we investigated gene expression patterns in poplar using public RNA-seq data (Luo *et al.*, 2019). The results suggested that *Potri.002G196200* responds to salt stress, and this gene was named as poplar *bZIP53* according to its sequence similarities (see below). To test this hypothesis, we treated 6-week plants with uniform growth with NaCl and conducted expression analyses. Since the stability of reference genes used for RT-qPCR is important for data confidence, we validated the stability of *ACTIN* and *UBQL* (Supplementary Table S1). Examination of the PCR specificity clearly indicated that the primers for the two genes could amplify single and solid bands in all the cDNA samples tested (Supplementary Fig. S1). These samples were prepared from RNA isolated from five different tissues (roots, stems, mature leaves, petioles, and young shoots) and from the stem base and roots of plants treated with 100 μM ABA or 150 mM NaCl in hydronic solution for different periods of time. Next, the quantification cycle (*C*_q_) values of *ACTIN* and *UBQL* were calculated using the same cDNA samples. The initial quantities of RNA used for cDNA synthesis were strictly measured to ensure that there were equal amounts of cDNA used for RT-qPCR. The ranges of *C*_q_ values for the two reference genes were less than 0.5 in the five different tissues and the six NaCl-treated samples, and were less than 1.0 in the six ABA-treated samples (Supplementary Fig. S1b). These results all suggested that the two genes had high stability and that they were therefore suitable for use in this study.

**Fig. 1.**
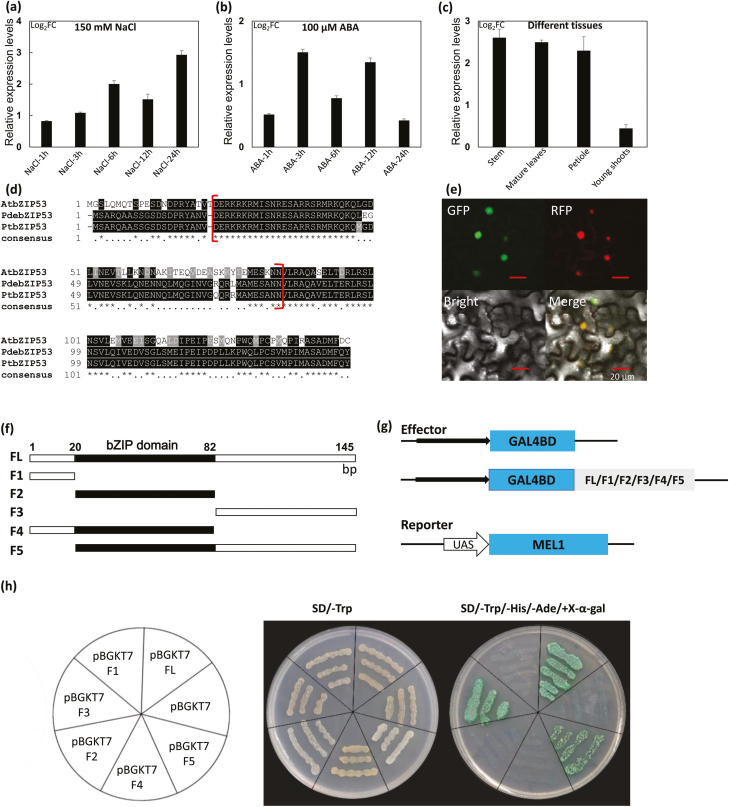
Characteristics of the poplar ‘NL895’ bZIP53 transcription factor. (a, b) Relative expression of *bZIP53* in response to treatment with (a) 150 mM NaCl and (b) 100 mM ABA for 1–24 h. The expression of untreated samples at 0 h was set as 1, and relative expression is presented as log_2_ values of the fold-change (FC). Plants were 6 weeks old with uniform growth. Data are means (±SE) of *n*=3 replicates. (c) Relative expression of *bZIP53* in different tissues. The expression of roots was set as 1, and relative expression is presented as log_2_FC values. Data are means (±SE) of *n*=3 replicates. (d) Alignment of the bZIP53 protein in Arabidopsis, *Populus trichocarpa*, and poplar ‘NL895’ (PdebZIP53). The red brackets indicate the location of the bZIP domains. (e) Subcellular localization of bZIP53, as determined by expression of *35S::bZIP53::GFP* or marker constructs in leaves of tobacco. RFP is a marker for localization in the nucleus. (f) Schematic diagram of the full-length (FL) poplar ‘NL895’ bZIP53 protein and the five fragments that were generated (F1–F5), which were used to construct effectors. (g) Schematic diagram of the effectors and the reporter. The boxes indicate genes and the black arrows indicate promoters. UAS is the upstream active sequence of *MEL1*, encoding melibiose. (h) Transactivation activity assays for the six effectors. Yeast strains were transformed with either the effectors or a negative control (pBGKT7), as shown in the diagram, and grown on SD/–Trp or SD/–Trp/–His/–Ade/+X-α-gal.

This RT-qPCR system was then used to investigate the expression of poplar *bZIP53* upon treatment with NaCl or ABA. Expression was clearly was increased by treatment with 150 mM NaCl (Fig. 1a) and expression in the stem base and roots was also increased by 2- to 3-fold after ABA treatment (Fig. 1b). We also examined the expression in different tissues, and found that it was much lower in the roots than in the aerial tissues (Fig. 1c).

The CDS of poplar *bZIP53* was cloned from roots of ‘NL895’ treated with NaCl. The full-length of the gene was 438 bp, and it encoded 145 amino acids (aa) (Supplementary Data S1). Phylogenetic analysis using Mega X (Kumar *et al.*, 2018) indicated that the protein was in the same sub-clade as, and showed the highest similarity with, Arabidopsis AtbZIP53 (Supplementary Fig. S2), and therefore we named it as PdebZIP53 (for NL895 which is hybrid of *P. deltoides* × *P. euramericana*); however, for clarity we refer to PdebZIP53 as ‘poplar bZIP53’ throughout the text. Comparing PdebZIP53 with AtbZIP53 and PtbZIP53 (from the reference genome of *P. trichocarpa*) at the protein level, PdebZIP53 shared 96% identity with PtbZIP53 and 61% identity with AtbZIP53. The bZIP domain was located at 21–82 aa, corresponding to the 61–246 bp position of the CDS (Fig. 1d).

Poplar bZIP53 was clearly located in the nucleus (Fig. 1e), suggesting that it was a transcription factor (TF). To investigate whether it possessed transactivation activity, the protein was divided into three parts according to the location of the bZIP domain. This resulted in the production of the following six fragments: FL (full-length), F1 (1–20 aa), F2 (21–82 aa), F3 (83–145 aa), F4 (1–82 aa), and F5 (21–145 aa) (Fig. 1f). These were fused with the GAL4 binding domain to generate a series of effector constructs (Fig. 1g), of which FL, F3, and F5 could activate the expression of the reporters (Fig. 1h). Since all three of these constructs contained the F3 fragment and the other constructs that lacked it could not activate the expression of reporters, we predicted that the activation domain was located at the 83–145 aa position in the poplar bZIP53 protein. Overall, the results suggested that poplar bZIP53 was a TF with transcription activity.

### Heterologous overexpression of poplar *bZIP53* inhibits the induction of ARs in Arabidopsis

Arabidopsis plants overexpressing poplar ‘NL895’ *bZIP53* or *bZIP53::GFP* in were generated, with overexpression of *bZIP53* being driven by the 2×35S promoter (Supplementary Fig. S3a) while *bZIP53::GFP* was driven by a single 35S promoter. Positive screening of single-copy transformation resulted in two OE-bZIP53 lines (OE-1 and OE-2) and one *35S::bZIP53:GFP* line (OE-b53-GFP) being selected for further analysis (Supplementary Fig. S3). Homozygous lines of the T_3_ generation were used for phenotypic assays. Since the expression of poplar *bZIP53* responds to salt treatment, we examined whether these transgenic plants showed increased salt tolerance. Seeds were sown on MS medium with 150 mM NaCl and no significant differences in growth between the WT and transgenic plants were observed after 15 d (Supplementary Fig. S3e). These results suggested that poplar bZIP53 was not involved in increasing/decreasing salt tolerance, although the gene did respond to salt stress.

**Fig. 2.**
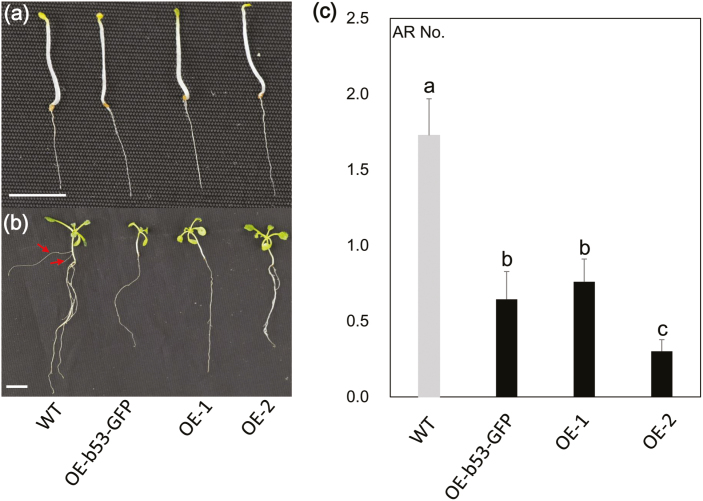
Phenotypes of Arabidopsis transgenic lines with heterologous overexpression (OE) of poplar *bZIP53*. (a) Representative images of the Arabidopsis lines after initial growth in the dark, and (b) after transfer to light for 10 d. The images show the wild-type (WT), OE-bZIP53-GFP (transformed with *35S::bZIP53::GFP*), and OE-1 and OE-2 (transformed with *2×35S*::*bZIP53*). The arrows indicate the induction of adventitious roots in the WT. Scale bars are 0.5 cm. (c) Mean number adventitious roots (ARs) for the lines shown in (b). Data are means (±SE) of *n*=50 replicates. Different letters indicate significant differences between means as determined using one-way ANOVA followed by Duncan’s multiple comparison test (*P*<0.05).

When seeds of the WT and the OE-1, OE-2, and OE-b53-GFP lines were grown in MS medium, we observed that the transgenic plants tended to form fewer ARs on the hypocotyl. We therefore performed a standard AR induction experiment (Sorin *et al.*, 2005) and found that after 10 d the three transgenic lines had significantly fewer ARs on the hypocotyl compared with the WT (Fig. 2). These results suggested that the poplar bZIP53 TF might negatively regulate AR development.

### Induced expression and overexpression of poplar *bZIP53* inhibit the development of ARs in poplar

To test whether *bZIP53* was involved in the regulation of AR development in poplar, two systems were employed for producing transgenic plants. First, similar to the OE lines of Arabidopsis, the construct *2×35S::bZIP53* (Supplementary Fig. S3A) was introduced into the genome of poplar ‘NL895’. This strategy enabled us to obtain more than 10 transgenic lines for a preliminary positive screening. Four lines, POE-1, POE-4, POE-5, and POE-6, were randomly selected for careful positive screening at the DNA, RNA, and protein levels (Supplementary Fig. S4). The results clearly showed that all four lines were stably transformed by the *2×35S::bZIP53* construct, and the expression of *bZIP53* increased 4- to 17.9-fold compared with the WT (Supplementary Fig. S4b). We examined three of the lines (POE-1, 4, 6) in more detail and they all showed fewer and shorter ARs at 12 d after the tissue-cultured microcuttings were inserted into woody plant medium (WPM) (Supplementary Fig. S5). The growth of all the OE lines was seriously inhibited, making it difficult to quickly obtain enough plants for further comprehensive phenotypic assays; however, the results clearly suggested that poplar *bZIP53* might negatively regulate AR formation and growth.

To quickly obtain more plant material for testing the functions of bZIP53 in poplar AR development and growth regulation, we employed a second strategy for producing transgenic plants with induced expression of *bZIP53*, using the variety ‘Shanxinyang’ for transformation due to its faster growth in WPM than ‘NL895’. The construct for induced expression of *bZIP53* is illustrated in Supplementary Fig. S6a. A total of 12 positive transgenic lines were screened by PCR confirmation (Supplementary Fig. S6b) and six plants harbouring *XVE::bZIP53* were randomly selected for growth in WPM with or without 10 μM oestradiol. The XVE system is a reliable and efficient chemical‐inducible system for regulating transgene expression in plants based on the regulatory region of the human estrogen receptor (Zuo *et al.*, 2000). After 8 d of treatment, RT-qPCR was used to examine the expression of *bZIP53* in the following four groups: ‘WT+ Water’ (WT lines growing in WPM), ‘WT+Estradiol’ (WT lines growing in WPM with 10 μM oestradiol), ‘T+Water’ (transgenic lines growing in WPM), and ‘T+Estradiol’ (transgenic lines growing in WPM with 10 μM oestradiol). The expression of *bZIP53* in the ‘T+ Oestradiol’ group was much higher than in the other three groups (Supplementary Fig. S6c). There were no visible differences between the growth of the WT lines in WPM with or without 10 μM oestradiol (Supplementary Fig. S6e). These results all indicated that the XVE transgenic system was effective in poplar.

**Fig. 3.**
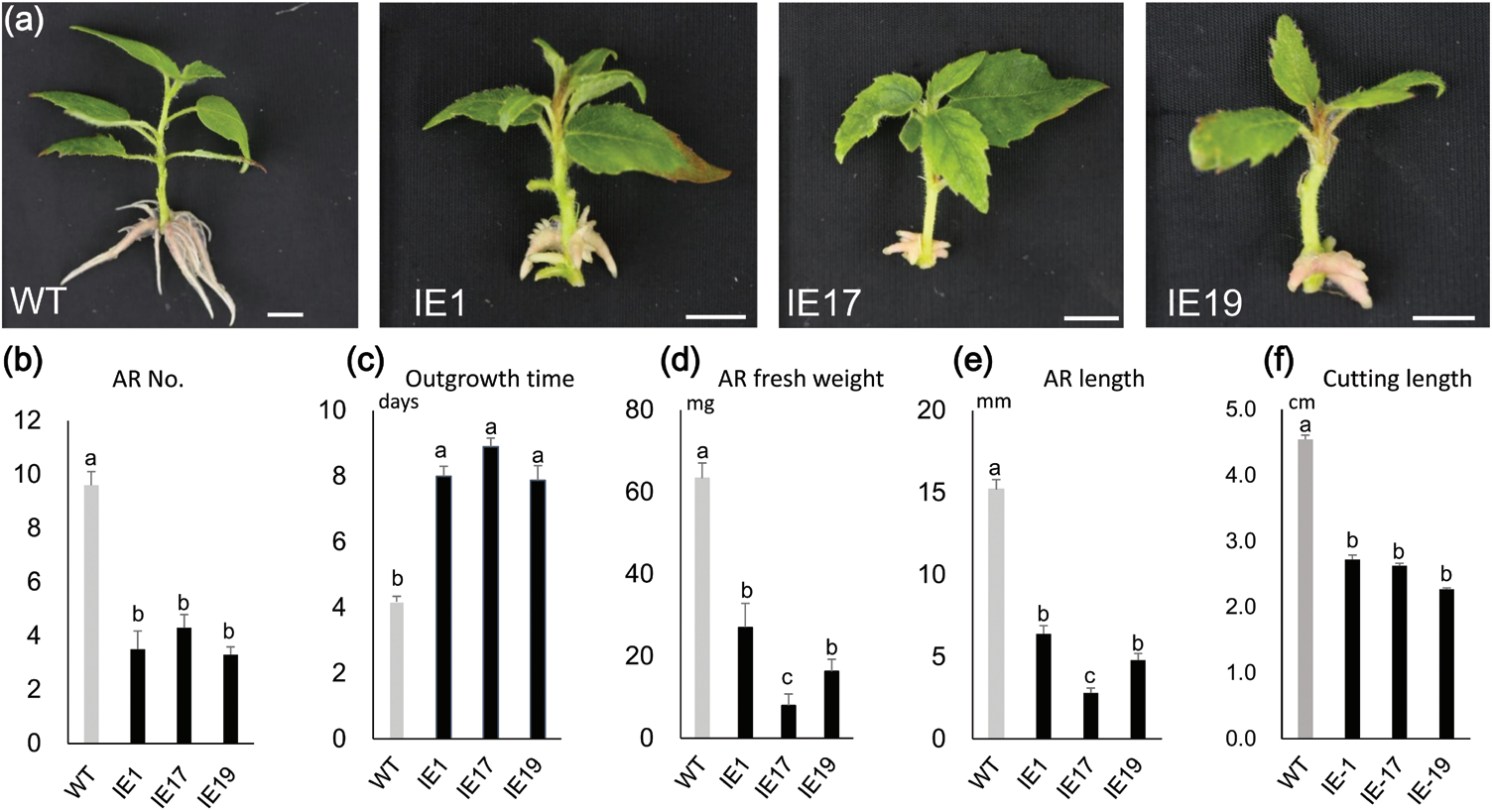
Phenotypes of transgenic poplar lines with induced overexpression of poplar *bZIP53*. Plants were grown in woody plant medium (WPM) for 12 d. (a) Representative images showing development of adventitious roots (ARs) in the wild-type (WT), and three induced-overexpression (IE) lines. The scale bars are 0.5 cm. (b) Mean number of ARs, (c) the mean amount of time taken for the first outgrowth of ARs to become visible, (d) mean fresh weight and (e) total length of ARs, and (f) stem length. The propagated plants were selected for uniform growth when they were transferred to WPM. Data are means (±SE) of *n*=30 replicates. Different letters indicate significant differences between means as determined using one-way ANOVA followed by Duncan’s multiple range test (*P*<0.05).

Three induced-expression lines (IE1, IE17, and IE19) were used for further observations. Tissue-cultured microcuttings with uniform growth were propagated in WPM with 10 μM oestradiol, and RT-qPCR assays after 4 d showed that the expression of *bZIP53* was highly induced in the IE lines compared with the WT (Supplementary Fig. S6d). The ARs for the three IE lines were significantly shorter than those of the WT at days 6 and 12 (Fig. 3a, e, Supplementary Fig. S7), and there were significantly fewer present at day 12 (Fig. 3b). The mean number of days required for the first AR outgrowth in the WT was significantly lower than that in the IE lines (Fig. 3c) whilst the fresh weight was greater in the WT on day 12 (Fig. 3d). The stem length was shorter in the IE lines (Fig. 3F). When the WT and the IE lines were grown in soil with application of 10 μM oestradiol solution to the stem bases for 12 d, the length of the ARs in the IE lines was also significantly shorter than that of the WT (Supplementary Fig. S8a, c), and the number of ARs and their fresh weight were both lower than in the WT (Fig. S8b, d). The stem length was also generally shorter in the IE lines (Supplementary Fig. S8e). Taken together, these results indicated that *bZIP53* negatively regulated AR development in poplar, and that induced expression of *bZIP53* also inhibited plant growth.

We also attempted to construct transgenic lines with reduced expression of *bZIP53*; however, the buds that generated all aborted during the selection procedure. This suggested that the bZIP53 TF is indispensable for poplar growth.

### Poplar *IAA4* is predicted as the downstream gene regulated by bZIP53

To further examine the mechanisms involved in the regulation of growth of ARs by bZIP53, transcriptome analysis based on RNA-seq was performed for the poplar WT and IE-*bZIP53* lines. In total, 2545 genes met the criteria for differentially expressed genes (DEGs), with 1535 down- and 1010 up-regulated genes in the IE lines (Supplementary Table S2). Among the DEGs there were a number of auxin-related genes. Because poplar *bZIP53* negatively regulated AR development and encoded a TF with transactivation activity, and because S-group bZIP TFs in Arabidopsis usually up-regulate their downstream genes, we mainly focused on the up-regulated genes in the IE lines.

**Fig. 4.**
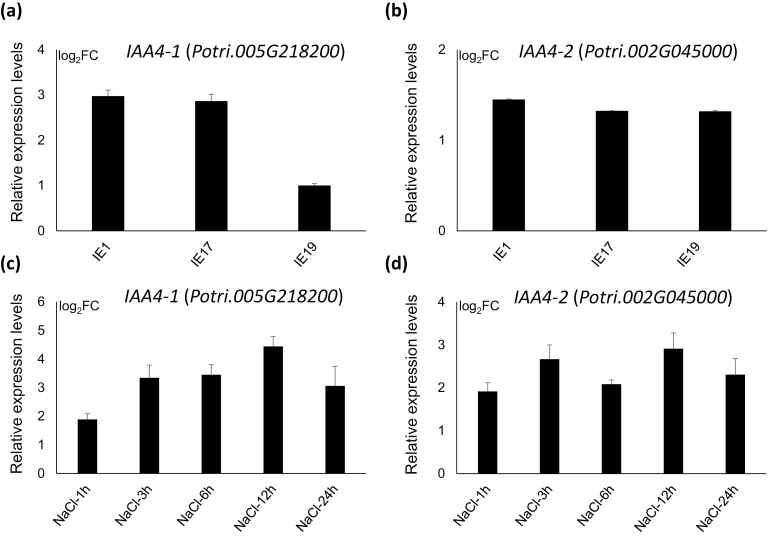
Expression of poplar *IAA4-1* and *IAA4-2* in transgenic lines with induced overexpression of *bZIP53*, and in wild-type plants treated with NaCl. Expression levels were determined using Rt-qPCR. (a, b) Relative expression of (a) *IAA4-1* and (b) *IAA4-2* in induced-overexpression (IE) lines. Expression in the wild-type (‘NL895’) was set as 1, and relative expression is presented as log_2_ values of the fold-change (FC). Plants were 12 d old and grown in WPM. (c, d) Relative expression of (c) *IAA4-1* and (d) *IAA4-2* in the wild-type following treatment with 150 mM NaCl for 1–24 h. The expression before treatment (0 h) was set as 1, and relative expression is presented as log_2_ values of FC. Data are means (±SE) of *n*=3 replicates.

Among the DEGs, two Arabidopsis genes homologous to *IAA3* and *IAA4* (members of Aux/IAA genes), namely *Potri.002G04500* and *Potri.005G218200*, showed increased expression in the IE samples. The phylogeny for Aux/IAA genes in poplar and Arabidopsis is illustrated in Supplementary Fig. S9, and the proteins of Potri.002G04500, Potri.005G218200, AtIAA3, and AtIAA4 were clearly grouped in the same sub-clade. In previous studies, AtIAA3 has been found to inhibit root growth by reducing polar auxin transport (Dello Ioio *et al.*, 2008). RT-qPCR assays indicated that, compared to the WT, the expression of *Potri.005G218200* was increased in IE1 and IE17 to a much higher degree than in IE19 (Fig. 4a). The expression of *Potri.002G04500* was increased more consistently across the three IE lines (Fig. 4b). The expression of poplar *bZIP53* in the roots and stem bases was induced by treatment with 150 mM NaCl (Fig. 1a) and therefore we also examined the expression of *Potri.005G218200* and *Potri.002G04500* under these conditions. RT-qPCR assays showed that the expression of both were induced by the NaCl treatment (Fig. 4c, d). The similar expression profiles for *Potri.005G218200*, *Potri.002G04500*, and *bZIP53* in response to NaCl suggested that their expression could be modulated by salt stress. The results also suggested that *bZIP53* might negatively regulate AR development in poplar by up-regulating *Potri.005G218200* and/or *Potri.002G04500*.

The full CDSs of *Potri.005G218200* and *Potri.002G04500* in ‘NL895’ were cloned, and they showed 60% similarity with AtIAA4 at the protein level (Supplementary Data S1). Since both the poplar proteins showed the highest similarity with AtIAA4, we named the genes poplar *IAA4-1* (*Potri.005G218200*) and *IAA4-2* (*Potri.002G04500*). To determine whether bZIP53 directly up-regulated the expression of *IAA4-1/2* in poplar, the ~1.6-kb promoter sequence for *IAA4*-1 and the ~1.3-kb promoter sequence for *IAA4-2* were cloned from ‘NL895’. In previous studies, the G-Box motif [(GTC)ACGT(GAT)]) has been reported to function as the key sequence element that interacts with the S1 group of bZIP TFs. Within the ~1.6-kb promoter sequence of *IAA4-1* there was a G-Box motif (CACGTG) located 590 bp upstream of the start translation codon ATG (Supplementary Data S1). Moreover, there was the same G-Box motif located 1209 bp upstream of the start translation codon ATG within the ~1.3 kb promoter sequence of *IAA4-2*. Altogether, this suggested that the poplar bZIP53 TF might up-regulate the expression of *IAA4-1* and *IAA4-2* via direct binding.

### Poplar bZIP53 TFs can directly bind to the promoter of *IAA4-1* and activate its expression

**Fig. 5.**
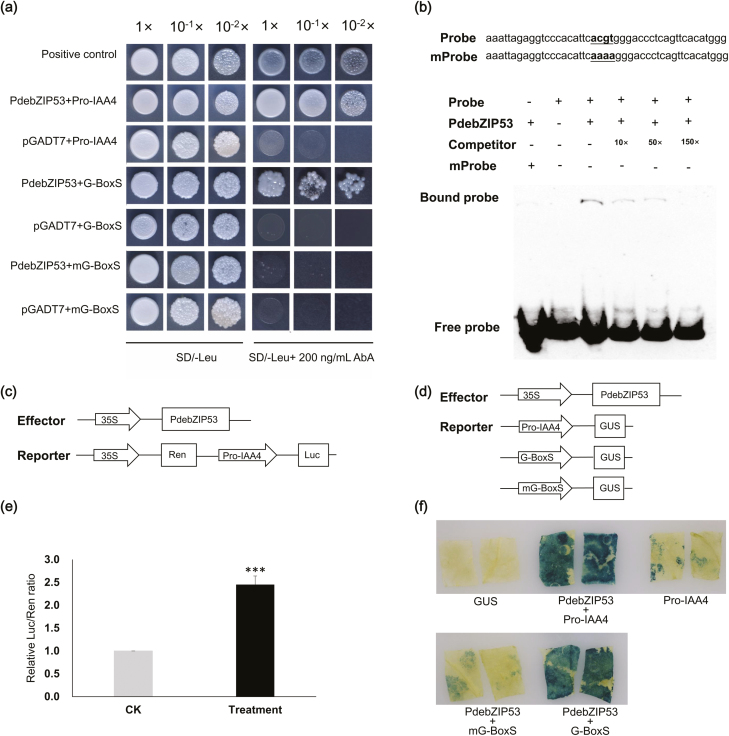
Protein–DNA interactions between poplar bZIP53 (PdebZIP53) and the *IAA4-1* promoter. (a) Yeast one-hybrid assays. Pro-IAA4 indicates the full promoter of *IAA4-1*. G-boxS indicates that the 340-bp sequence includes the mG-Box motif in IAA4-Pro. mG-boxS indicates the mutated form of G-boxS (identical sequence to G-BoxS except for the 6-bp G-Box). The serial dilutions are indicated at the top. (b) Electrophoretic mobility shift assay. The probe sequence occurs in IAA4-Pro and harbours the G-Box motif, while in the mutated probe (mProbe) the four core bases of the G-Box were substituted with ‘aaaa’. ‘+’ and ‘–’ indicate that the reagents were present or absent in the lane during protein electrophoresis. The bound probe indicates an interaction between bZIP53 and the probe. (c) Schematic diagram for the construction of the effectors and reporters used in the dual-luciferase reporter assay. Arrows indicate promoters and boxes indicate genes. (d) Schematic diagram for the construction of the effectors and reporters used in the GUS assay. (e) Results of the dual-luciferase assay illustrated in (c), showing the Luc/Ren ratios for co-expression of the empty effector control (CK, without *bZIP53* inserted into the effector plasmid) and for co-expression of the effector and reporter in (Treatment). (f) Results of the GUS staining illustrated in (d).

The full-length promoter sequence of *IAA4-1* (designated as Pro-IAA4), a 340-bp sequence containing the G-Box within Pro-IAA4 (designated as G-BoxS), and the 6-bp G-Box motif were substituted with ‘CAAAAG’ (designated as mG-boxS) and used for Y1H assays (all sequences are listed in Supplementary Data S1). Using this system, we found that poplar bZIP53 could interact with Pro-IAA4 and G-BoxS in yeast, while there was no interaction between bZIP53 and mG-BoxS (Fig. 5a). Electrophoretic mobility shift assays (EMSAs) were further employed to test the interaction between bZIP53 and the G-Box motif. The 45-bp sequence containing the G-Box motif within the promoter of Pro-IAA4 was used as the probe (Probe), while the G-Box motif was substituted with ‘CAAAAG’ using a mutated probe (mProbe) (Fig. 5b). The poplar bZIP53 TF was clearly bound to the Probe, while there was only a very weak binding signal for bZIP53/mProbe. This clearly indicated that the poplar bZIP53 TF could directly bind the promoter of IAA4, and that the G-Box motif was the binding site. In addition, the very weak binding signal for bZIP53/mProbe in the EMSA might suggest that some other similar sequence of the G-Box can form weak interactions with bZIP53, for example the ‘CACATT’ upstream of the G-Box differed from it by only one base, and this sequence might form weak interactions with the bZIP53 TF.

We then tested the interaction between bZIP53 and the promoter of *IAA4-1* using dual-luciferase reporter and GUS staining assays. Using the 35S promoter to drive the expression of *bZIP53* (effector) and Pro-IAA4 to drive the expression of *LUC* (reporter) (Fig. 5c), we found that poplar bZIP53 could activate the reporter when co-expressed with the effector and the reporter in tobacco leaves (Fig. 5e). This suggested that the bZIP53 TF could up-regulate the expression of poplar *IAA4-1 in vivo*. Another transient co-expression system was also employed to test this regulation (Fig. 5d, f). Co-expression of *35S::bZIP53* and *Pro-IAA4::GUS* was induced in tobacco leaves, and the *35S::bZIP53*/*Pro-IAA4::GUS* and *35S::bZIP53*/*G-BoxS::GUS* pairs showed strong GUS staining, while the control (empty *1301S/Pro-IAA4::GUS*) showed no staining (Fig. 5f). Interestingly, slight GUS staining was also observed for *35S::bZIP53*/*mG-BoxS::GUS* and *Pro-IAA4::GUS* co-expression. For *35S::bZIP53*/*mG-BoxS::GUS*, this could also be attributable to the weak interaction between bZIP53 and mG-boxes, similar to the results of the Y1H assays. The slight GUS staining for *Pro-IAA4::GUS* might suggest that the expression of tobacco *bZIP53* was induced and that this gene could promote the expression of *Pro-IAA4::GUS*. This could explain why there was only a ~2.5-fold relative Luc/Ren ratio (Fig. 5e). Because the promoters of *IAA4-1* and *IAA4-2* both possessed a G-box, we only examined the direct interaction between bZIP53 and the *IAA4-1* promoter. Overall, the results suggested that the poplar bZIP53 TF could promote the expression of poplar *IAA4-1/2* through direct regulation.

### Induced expression of *IAA4-1/2* inhibits AR development in poplar

**Fig. 6.**
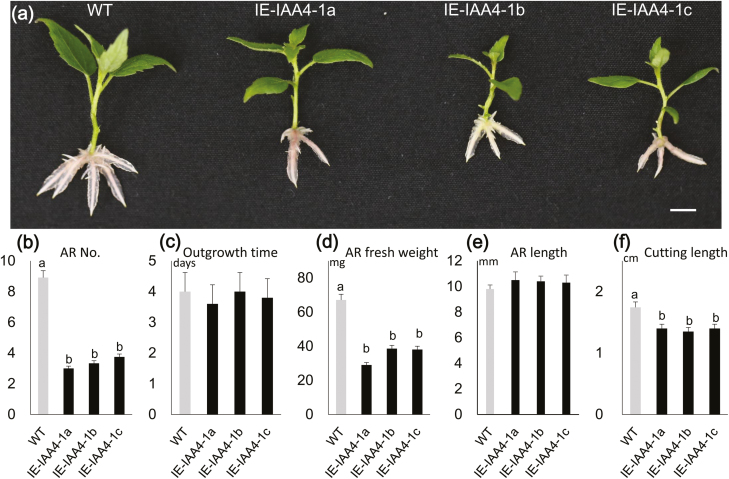
Phenotypes of transgenic poplar lines with induced overexpression of poplar *IAA4-1*. Plants were grown in woody plant medium (WPM) for 12 d. (a) Representative images showing development of adventitious roots (ARs) in the wild-type (WT), and three induced-overexpression (IE) lines. The scale bars are 0.5 cm. (b) Mean number of ARs, (c) the mean amount of time taken for the first outgrowth of ARs to become visible, (d) mean fresh weight and (e) total length of ARs, and (f) stem length. The propagated plants were selected for uniform growth when they were transferred to WPM. Data are means (±SE) of *n*=30 replicates. Different letters indicate significant differences between means as determined using one-way ANOVA followed by Duncan’s multiple range test (*P*<0.05).

To examine whether *IAA4-1/2* were involved in the regulation of AR development in poplar, the constructs *XVE*::*IAA4-1* and *XVE*::*IAA4-2* were introduced into the genome of the variety ‘Shanxinyang’. More than 20 positive transgenic lines were generated for each gene and three IE lines for each were selected for positive transgenic confirmation (Supplementary Fig. S10). The lines for *IAA4-1* were designated as IE-IAA4-1a, IE-IAA4-1b, and IE-IAA4-1c, while those for *IAA4-2* were designated as IE-IAA4-2a, IE-IAA4-2b, and IE-IAA4-2c. When the lines were grown in WPM, the number of ARs for all six IE lines were significantly lower than those of the WT at days 6 and 12 (Supplementary Fig. S11; Fig. 6a, Supplementary Fig. S12), but the length of the ARs showed no differences at day 12. The time required for emergence of ARs showed no differences between the WT and the IE-IAA4-1 and IE-IAA4-2 lines (Fig. 6c, Supplementary S12c), and there were also no differences in the length of ARs (Fig. 6e, Supplementary S12e). The AR fresh weight was lower in the *IAA4-1/2* IE lines than in the WT lines (Fig. 6d, Supplementary S12d). The stem length was significantly shorter in the *IAA4-1* IE lines than in the WT (Fig. 6f); however, whilst it was also somewhat shorter in the *IAA4-2* IE lines, the difference with the WT was not significant (Supplementary S12f). These results indicated that the induced expression of *IAA4-1/2* in poplar could reduce the number and fresh weight of ARs without affecting their length, and that there was also a general reduction in stem length. Compared with AR growth in poplar lines with induced expression of *bZIP53* (Fig. 3, Supplementary Figs S5, S7), the degree of inhibition of development was weaker when expression of *IAA4-1/2* was induced.

In order to further investigate the differences in AR growth inhibition between the lines with induced expression of *bZIP53* and *IAA4-1/2*, the transgenic lines IE1, IE17, IE-IAA4-1a, and IE-IAA4-2a were propagated in WPM for 6 d, which enabled ARs to be induced. The cuttings were then transferred to WPM with 10 μM oestradiol for 6 d. The AR lengths of the two *bZIP53* lines were still reduced, while the two *IAA4* lines showed AR lengths that were more similar to those of the WT (Supplementary Fig. S13). The mean AR lengths were 1.2±0.2 cm for the WT, 0.5±0.1 cm for IE1, 0.3±0.1 cm for IE17, 1.1±0.2 cm for IE-IAA4-1a, and 1.1±0.1 cm for IE-IAA4-2a. The mean number of ARs ranged from 6.0–8.5 and there was no significant differences among the lines. These results suggested that bZIP53 could inhibited AR growth in terms of both numbers and length, while IAA4 mainly affected the number. We were therefore able to conclude that bZIP53 and IAA4-1/2 formed a regulatory module, and that this module was involved in the negative regulation of AR development in poplar.

### Exogenous auxin application partially relieves the inhibition of AR growth caused by induced expression of *bZIP53* and *IAA4-1/2*

We next examined whether the growth inhibition of ARs caused by induced expression of *bZIP53* and *IAA4-1/2* in poplar could be affected by exogenous application of auxin. The WT and the transgenic lines IE1, IE17, IE-IAA4-1a, and IE-IAA4-2a were propagated in WPM with 10 μM oestradiol and either with or without 1 mg l^–1^ IAA for 8 d. AR growth in the WT was inhibited by the addition of IAA whereas all the transgenic lines of *bZIP53* and *IAA4-1/2* showed better AR growth with IAA, and this was generally somewhat better than that observed in the WT (Supplementary Fig. S14). However, the addition of 1 mg l^–1^ IAA did not eliminate all the inhibition caused by the induced expression of *bZIP53* or *IAA4-1/2*. We tested several other concentrations of IAA, but 1.0 mg l^–1^ showed the most effect. Overall, the results indicated that exogenous auxin application could partially relieve the AR growth inhibition caused by induced expression of *bZIP53* and *IAA4-1/2*, and they also suggested that the bZIP53–IAA4-1/2 regulatory module could interact with, or be involved in, pathways regulated by auxin.

## Discussion

### A model for the regulation of AR development by the bZIP53–IAA4 module

**Fig. 7.**
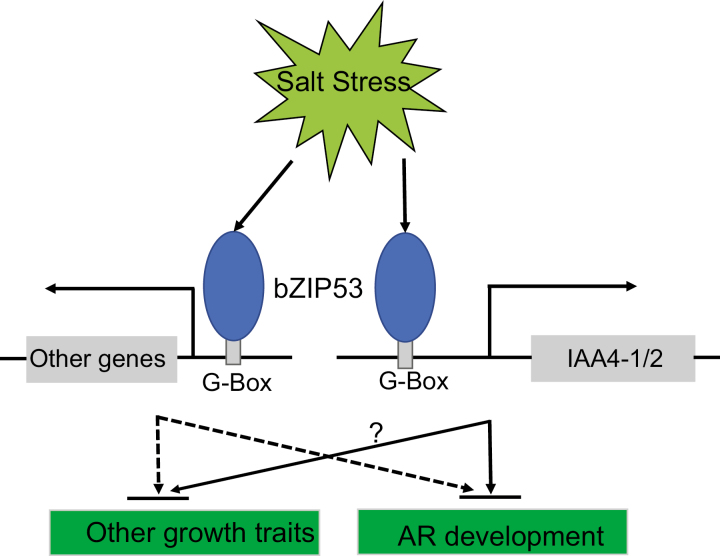
A schematic model for the regulation of development of adventitious roots (ARs) in poplar under the control of *bZIP53* and *IAA4*. Solid arrows indicate regulation as demonstrated in this study whilst dashed lines indicate predicted regulation. Salt stress activates the expression of *bZIP53*, and the bZIP53 protein in turn activates the expression of *IAA4-1/2*. The *IAA4-1/2* proteins then inhibit the development of ARs. Other plant growth traits are also inhibited by the bZIP53–IAA4 module.

Our results lead us to propose a regulatory model for the development of adventitious roots (ARs), in which the G-Box was forms the bridge between bZIP53 and *IAA4-1/2* (Fig. 7). This is consistent with previous reports (Foster *et al.*, 1994; Sibéril *et al.*, 2001). *IAA4-1/2* belongs to the Aux/IAA gene family, the proteins of which are degraded by the ubiquitin-ligase SCF^TIR1^ after exposure to auxin. This degradation leads to the expression of downstream auxin-responsive factors (ARFs) (Gray *et al.*, 2001; Zenser et al., 2001; Baster *et al.*, 2013). In Arabidopsis, IAA3 has been found to negatively regulate the auxin transporter PIN proteins and to redistribute auxin (Dello Ioio *et al.*, 2008). According to our phylogenetic analysis, poplar IAA4-1/2 were grouped in the same sub-clade as AtIAA3/4 (Supplementary Fig. S9), suggesting that they may have similar functions. We therefore speculate that poplar IAA4-1/2 also play roles in the regulation of auxin redistribution and hence affect AR development. Further studies are needed confirm this hypothesis.

Comparison of the phenotypes between plants with induced expression of *bZIP53* and *IAA4-1/2* indicated that the inhibition of AR development was clearly different (Figs 3, 6, Supplementary Figs S11, S12). The AR growth of the three IE-*bZIP53* lines was largely inhibited in terms of number, length, and biomass, whereas in the IE-*IAA4-1/2* lines the inhibition was in the form of number and biomass but not length. In addition, in terms of biomass the IE-*IAA4-1/2* lines showed less of a reduction than the IE-*bZIP53* lines. There are two possible explanations for this. First, *IAA4-1* and *IAA4-2* have two different gene loci that can be transcribed individually. In the IE-*bZIP53* lines, both of the genes were up-regulated, whereas only one was up-regulated in IE-*IAA4-1* and IE-*IAA4-2*. The different amounts of *IAA4* transcripts in the IE-*bZIP53* and IE-*IAA4-1/2* lines might therefore have resulted in the different levels of AR inhibition. Second, there are likely to be other downstream genes of bZIP53, and they will probably also negatively regulate AR development. In our RNA-seq results for IE-*bZIP53* lines, some auxin-related genes were also highly upregulated, for example the homologs of Arabidopsis GH3.17 (Supplementary Table S2).Some GH3 proteins have been reported to conjugate auxin and thus negatively regulate root or AR development (Staswick *et al.*, 2005; Gutierrez *et al.*, 2012). Although we were not able to determine which of these two possibilities contributed to the different degrees of AR inhibition, we include the second one in our regulatory model (Fig. 7).

Although our model fits the experimental results very well, it should be noted that we only used transgenic lines with induced-expression and overexpression for our phenotype assays, and the lack of reduced-expression lines for both *bZIP53* and *IAA4-1/2* could somewhat weaken its confidence. In our future studies we hope to add more evidence for the model and to identify other genes that are regulated by bZIP53.

### Poplar bZIP53 negatively regulates plant growth

In Arabidopsis, the bZIP53 transcription factor (TF) shows a response to low-energy and salt stresses (Dietrich *et al.*, 2011; Hartmann *et al.*, 2015), and *AtbZIP53* overexpression can inhibit plant growth. In our study, we also found strong inhibition of growth in the IE- and OE-*bZIP53* lines. The stem length was shorter in the OE-*bZIP53* lines (Figs. 3, 6, Supplementary Fig. S8), and heterologous overexpression of poplar *bZIP53* in Arabidopsis also inhibited growth (Fig. 2b). In the IE *IAA4-1/2* lines, both genes seemed to result in some inhibition of plant growth (Fig. 6, Supplementary Fig. S12), but the degree of inhibition was much weaker than that observed for *bZIP53*. Although we are not able to exclude the possibility that the inhibition of plant growth in the transgenic lines could be attributable to the weaker growth of ARs, our results may still suggest that poplar bZIP53 negatively regulates plant growth by modulating the expression of other genes. In Arabidopsis, salt-induced expression of *AtBZIP53* and *AtbZIP1* can regulate primary C and N metabolism and thus affect plant growth (Hartmann *et al.*, 2015), and the genes regulated downstream of AtbZIP53 include a number that are related to energy. In our RNA-seq results for poplar *bZIP53*, there were also a number of energy-related genes that were up-regulated in the induced-expression lines (Supplementary Table S2). We therefore speculate that *IAA4-1/2* were not the only downstream genes regulated by bZIP53 in poplar. There are likely to have been a number of other genes involved in the inhibition of plant growth, either directly or indirectly up-regulated by bZIP53 (Fig. 7)

### Poplar bZIP53 may play roles in integrating abiotic stress responses and in the control of plant growth

Plants are exposed to numerous stresses throughout their life cycle, and the deployment of resistance responses results in the consumption of additional energy. To meet this requirement, plants usually balance energy allocation in a way that leads to decreased growth, as we found in a previous integrated transcriptome study in poplar (Luo *et al.*, 2019). In our current study, the inhibition of stem growth and AR development might have been the result of this energy re-assignment, which resulted in more energy being allocated to the resistance response to salt stress. We speculate that bZIP53 re-assigned this energy by directly modulating auxin-related genes, for example *IAA4-1/2* and *GH3.17*. We therefore conclude that poplar bZIP53 may play a role in integrating abiotic stress responses and auxin-mediated control of plant growth in poplar. The inhibition of AR growth in the *bZIP53*-IE and *IAA4-1/2*-IE transgenic lines could be partially relieved by exogenous application of IAA (Supplementary Fig. S14), thus providing evidence for a link between auxin signalling and *bZIP53*. Several genes and micorRNAs have also been reported to link auxin signalling with abiotic stresses (Ke *et al.*, 2015; Dash *et al.*, 2017; He *et al.*, 2018). Moreover, AtbZIP11 links low-energy signalling to auxin-mediated control of primary root growth in Arabidopsis (Weiste *et al.*, 2017). Both bZIP53 and bZIP11 belong to the S-group of bZIP TFs. Thus, poplar bZIP53 plays similar roles to bZIP11 in Arabidopsis, making our model of regulation consistent with the results of previous studies.

## Supplementary Data

Supplementary data are available at *JXB* online.

Fig. S1. Confirmation of the stability of the reference genes *ACTIN* and *UBQL*.

Fig. S2. The phylogeny of bZIP transcription factors in Arabidopsis and *P. trichocarpa*.

Fig. S3. Positive transgenic screening of *bZIP53*-overexpressing lines of Arabidopsis, and results of NaCl treatments.

Fig. S4. Positive transgenic screening of *bZIP53*-overexpressing lines of poplar ‘NL895’.

Fig. S5. Adventitious root development in poplar *bZIP53*-overexpressing transgenic lines in woody plant medium.

Fig. S6. Positive transgenic screening of induced overexpression in *bZIP53* lines of poplar ‘Shanxinyang’.

Fig. S7. Adventitious root development in transgenic induced-overexpression *bZIP53* lines in woody plant medium.

Fig. S8. Phenotypes of poplar lines with induced overexpression of *bZIP53* grown in soil.

Fig. S9. Phylogenetic analysis of Aux/IAA genes in Arabidopsis and *P. trichocarpa*.

Fig. S10. Positive transgenic screening of induced overexpression in *IAA4-1/2* lines in poplar ‘Shanxinyang’.

Fig. S11. Adventitious root development in transgenic induced-overexpression *IAA4-1/2* lines in woody plant medium.

Fig. S12. Phenotypes of poplar lines with induced overexpression of *IAA4-2* in woody plant medium.

Fig. S13. Adventitious root development for transgenic induced-overexpression lines of poplar in woody plant medium with addition of oestradiol.

Fig. S14. Adventitious root development for transgenic induced-overexpression lines of poplar in woody plant medium with or without addition of IAA.

Table S1. Primers used in this study.

Table S2. Differentially expressed genes in induced-expression *bZIP53* lines.

Data S1. DNA sequences.

eraa096_suppl_Supplementary_Table

eraa096_suppl_Supplementary_Figures

eraa096_suppl_Supplementary_Data_S1

eraa096_suppl_Supplementary_Data_S2
