# Supplementary material for: The bZIP53–IAA4 module inhibits adventitious root development in Populus
Source: J Exp Bot. 2020 Feb 20;71(12):3485–98. doi: 10.1093/jxb/eraa096 (PMC7307859; doi:10.1093/jxb/eraa096)
Supplement: eraa096_suppl_Supplementary_Figures [file eraa096_suppl_supplementary_figures.pdf]

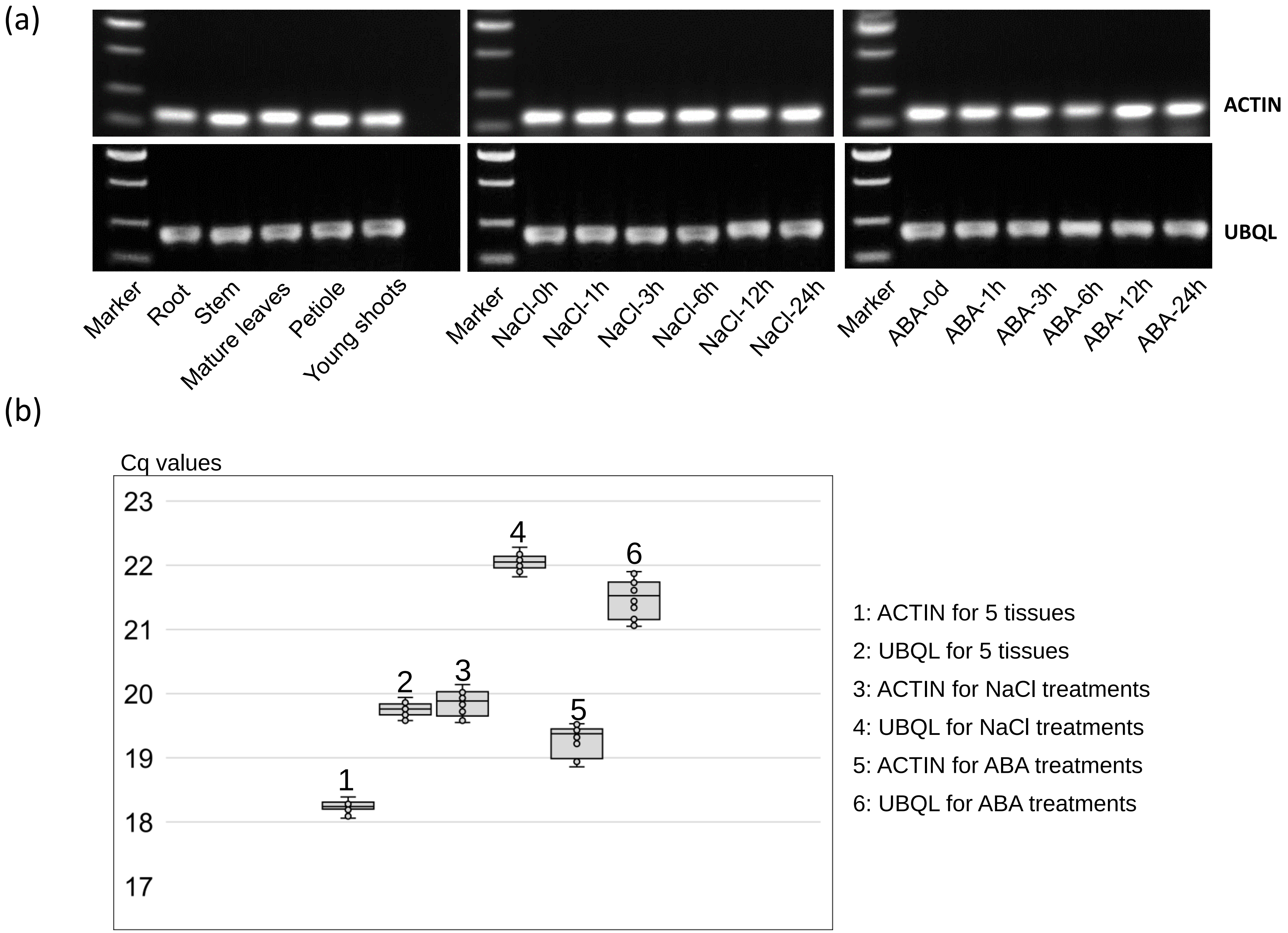

Figure S1. Confirmation of the stability for the reference genes *ACTIN* and *UBQL*.
(a)PCR speciality confirmation by agarose gel electrophoresis. The sizes for DNA marker were 750, 500, 250 and 100 bp, respectively (from above to bottom). cDNA was synthesized from RNAs isolated five different tissues (including roots, stems, mature leaves, petiole and young shoots), stem base and roots (under the hydronic solution) treated with 100 μM ABA and 150 mM NaCl. The 6-week plants with uniform growth status were used for plant materials. (b) The quantification cycle (Cq) values of *ACTIN* and *UBQL* for the above cDNA samples. Equal amounts of cDNA for the same type of samples were used for RT-qPCR.

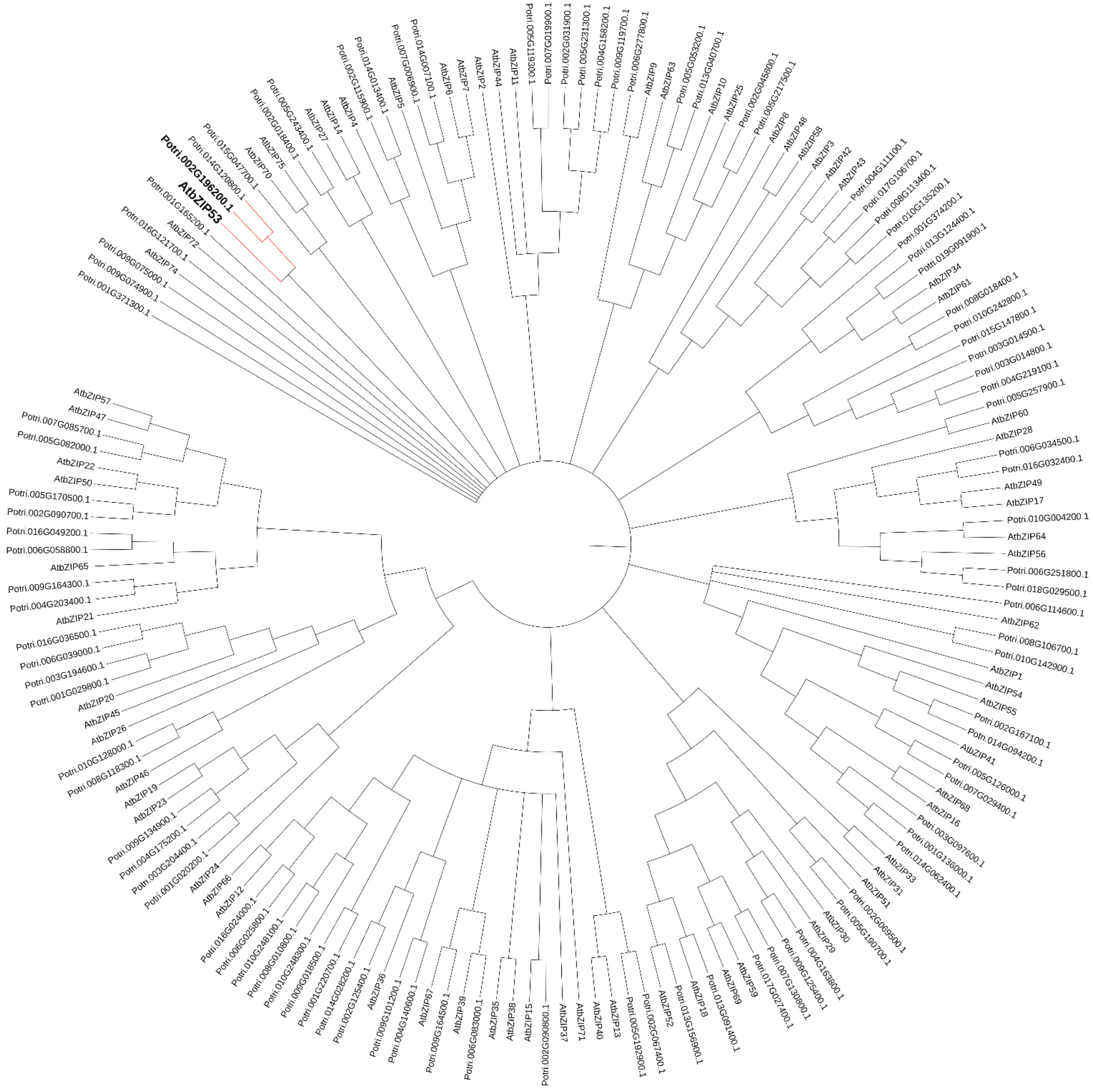

Figure S2. The phylogeny of bZIP transcription factors in *A. thaliana* and *P. trichocarpa*
Protein sequences were used to construct the phylogeny with the neighbour-joining method and 1000 bootstrap replications.

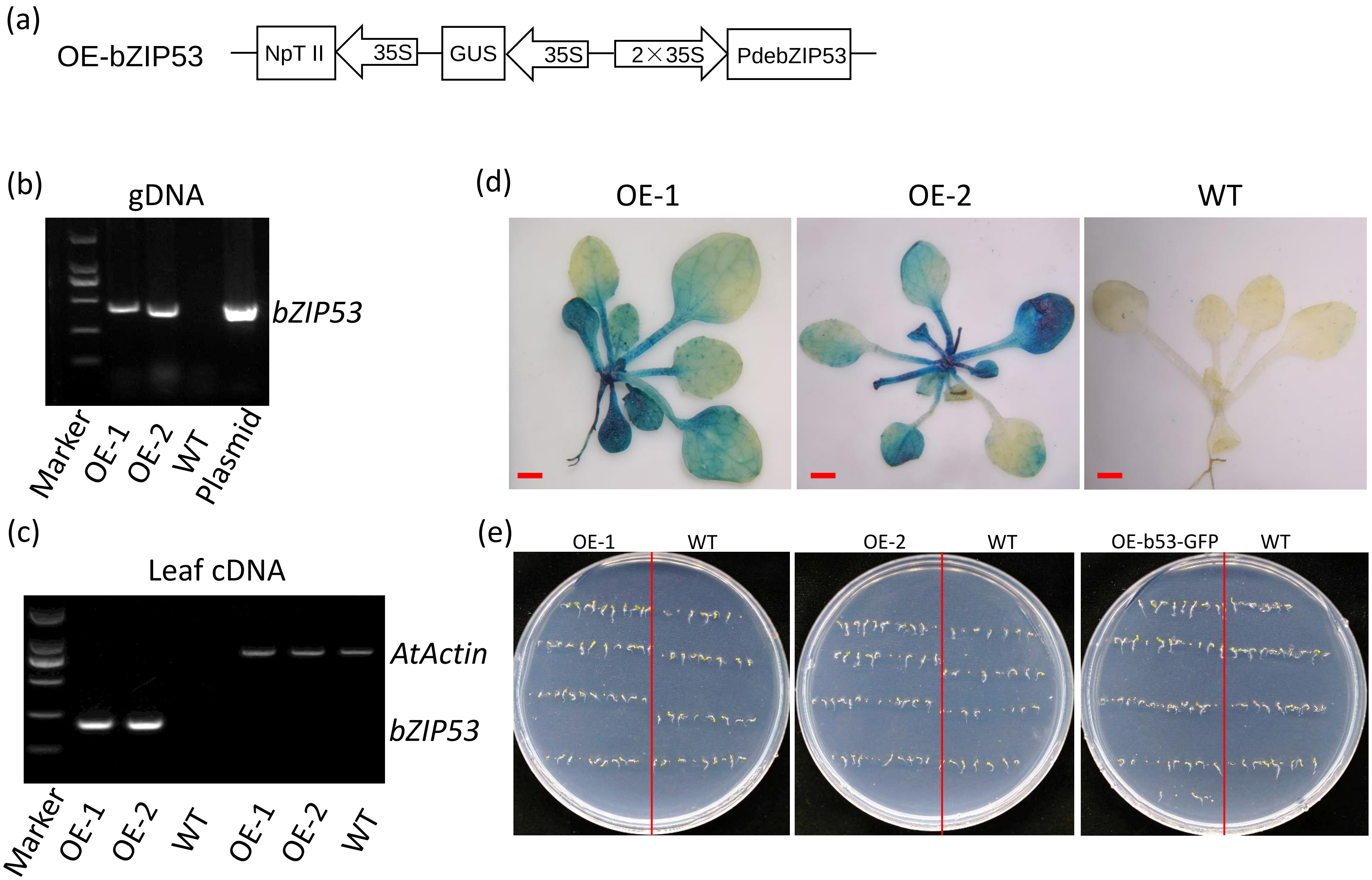

Figure S3. Positive transgenic screening of overexpressed *bZIP53* lines in *A. thaliana* and NaCl-treated assay Schematic diagram for the construction of the OE-bZIP53 vector was shown in (a). Three screenings were performed at the DNA, RNA and protein levels, as shown in (b), (c) and (d), respectively. (e) Transgenic *bZIP53* lines in *A. thaliana were* treated with 150 mM NaCl for 10 days. Sequence between RB (right border) and LB (left border) of the binary vector 2301S was illustrated in (a). Npt II represents genes encoding kanamycin resistance. PCR amplification of bZIP53 using the primer pair 1301s-testF/bzip531R was visualized in (b). The sizes for DNA markers from bottom to top are 100, 250, 500, 750, 1000 and 2000 bp. The bars in (d) are equal to 1 mm.

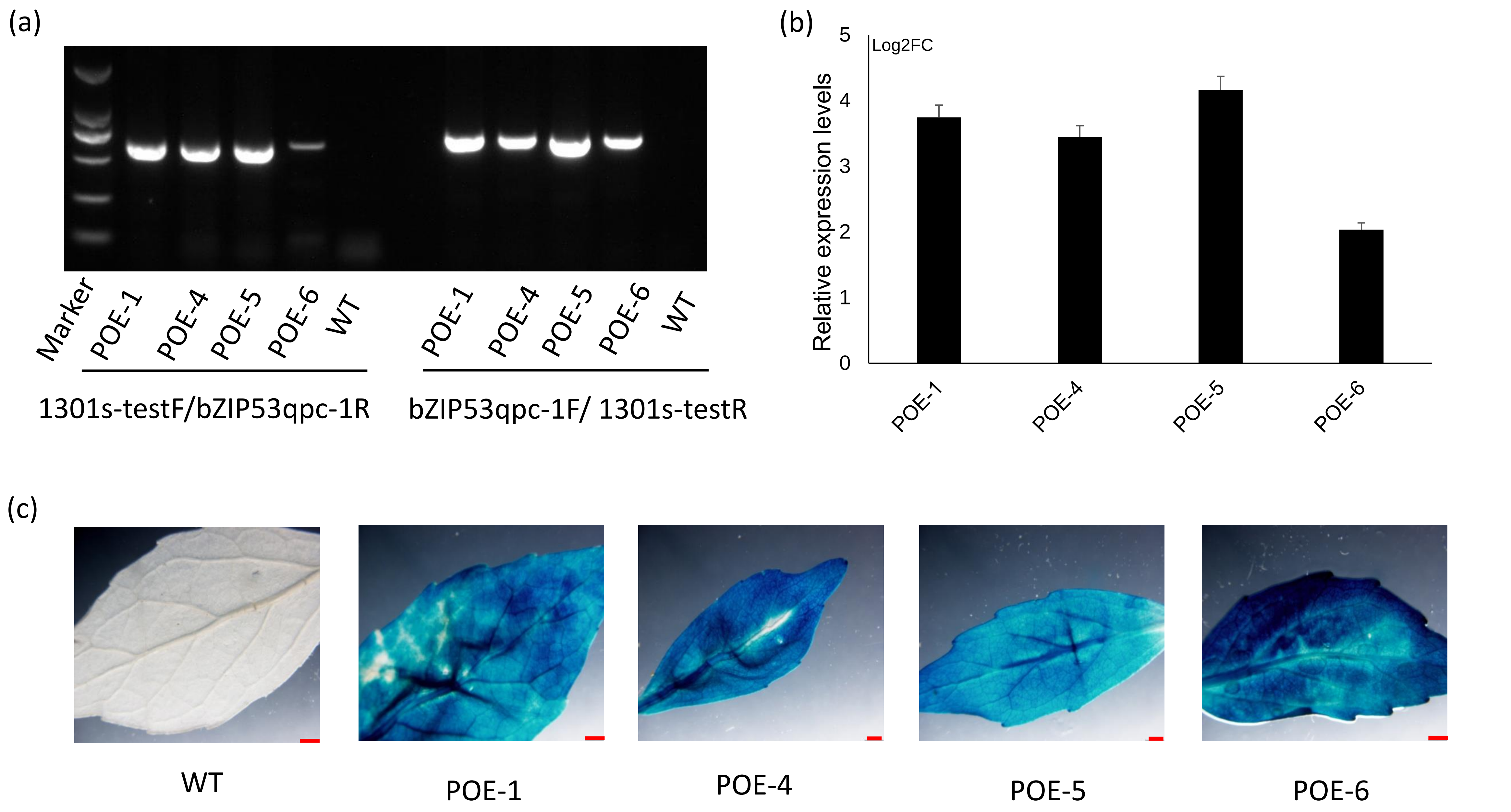

Figure S4. Positive transgenic screening of overexpressed *bZIP53* transgenic lines in poplar 'NL895'
Three screenings were performed at the DNA, RNA and protein levels, as shown in (a), (b) and (c), respectively. The construct of OE-bZIP53 was identical to the production of overexpressed *bZIP53* transgenic lines in *A. thaliana* and it was illustrated in Fig. S2a. PCR amplifications of *bZIP53* using the primer pair 1301s-testF/bZIP53qpc-1R (left) and bZIP53qpc-1F/1301s-testR (right) were visualized in (a). The sizes for DNA markers from bottom to top are 100, 250, 500, 750, 1000 and 2000 bp. The relative expression (represented by log_2FC) of *bZIP53* in the 4 transgenic to WT poplar lines were in (b). The expression of WT lines was set as 1. The calculation of log_2FC is similar to Fig. 1. The bars in (c) are equal to 0.1 cm.

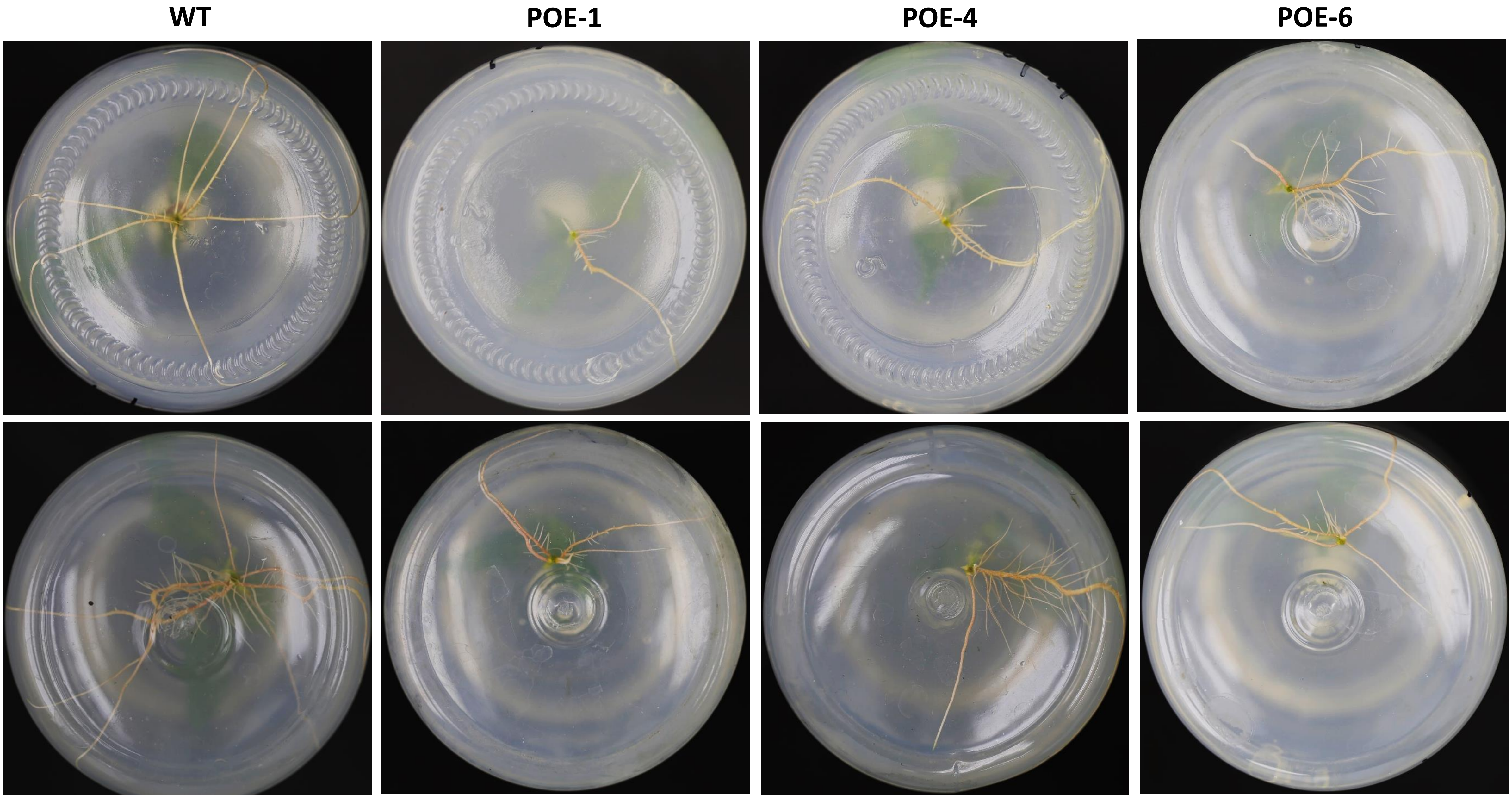

Figure S5. Adventitious root development in overexpressed *bZIP53* transgenic lines in WPM
Cuttings of WT and overexpressed *bZIP53* lines were grown in WPM for 12 days. The cuttings initially showed the same growth status. The diameter of the tissue culture bottles was 7.0 cm.

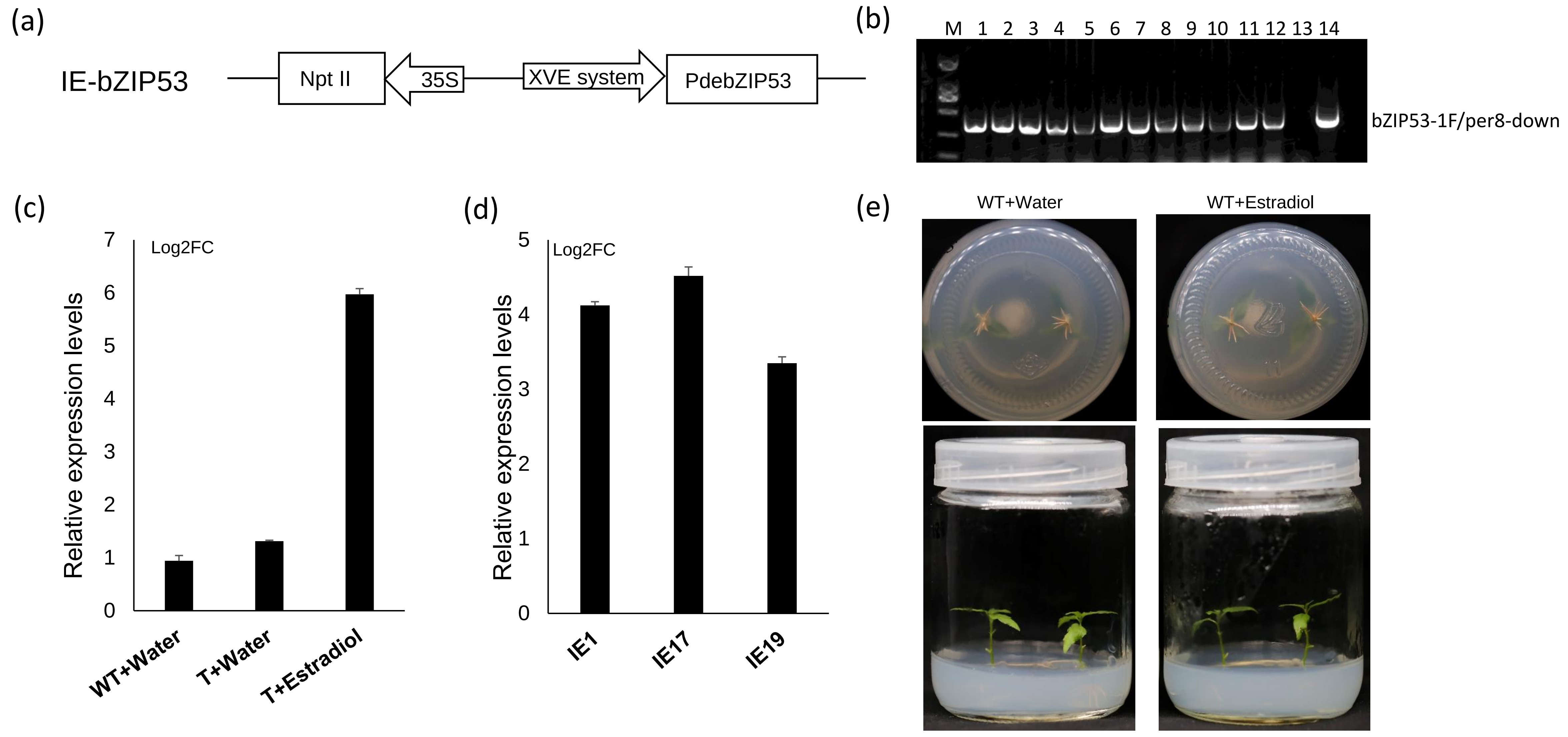

Figure S6. Positive transgenic screening of induced overexpression of *bZIP53* transgenic lines in poplar 'Shanxinyang' Schematic diagram for the construction of the IE-bZIP53 vector. XVE system represents that gene downstream of XVE can be activated by oestradiol treatment. (b) PCR confirmed the 12 positive transgenic lines. M indicates DNA ladder, and the sizes are the same as in the above figures. Lanes 1 to 12 indicate positive transgenic lines 1 to 12, while lanes 13 and 14 indicate WT and plasmid harbouring *bZIP53* CDSs. (c) qRT-PCR assay for the expression of *bZIP53* under different conditions. WT indicates the wild type of "Shanxinyang"; T indicates a mixture of 3 transgenic "Shanxinyang" root samples; WT+Water and WT+Estradiol indicate WT plants growing in water and solution with 10 μM oestradiol, respectively. The relative expression value (represented by log2FC) of *bZIP53* in WT+Estradiol was set as 1. (d) RT-qPCR assay for the expression of *bZIP53* in WT and 3 IE lines. The relative expression value (represented by log_2FC) of *bZIP53* in WT lines was set as 1. The calculation of log2FC is similar to Fig. 1. (e) WT lines were propagated in WPM with or without10 μM oestradiol for 6 days. The initial cuttings were in the same growth status.

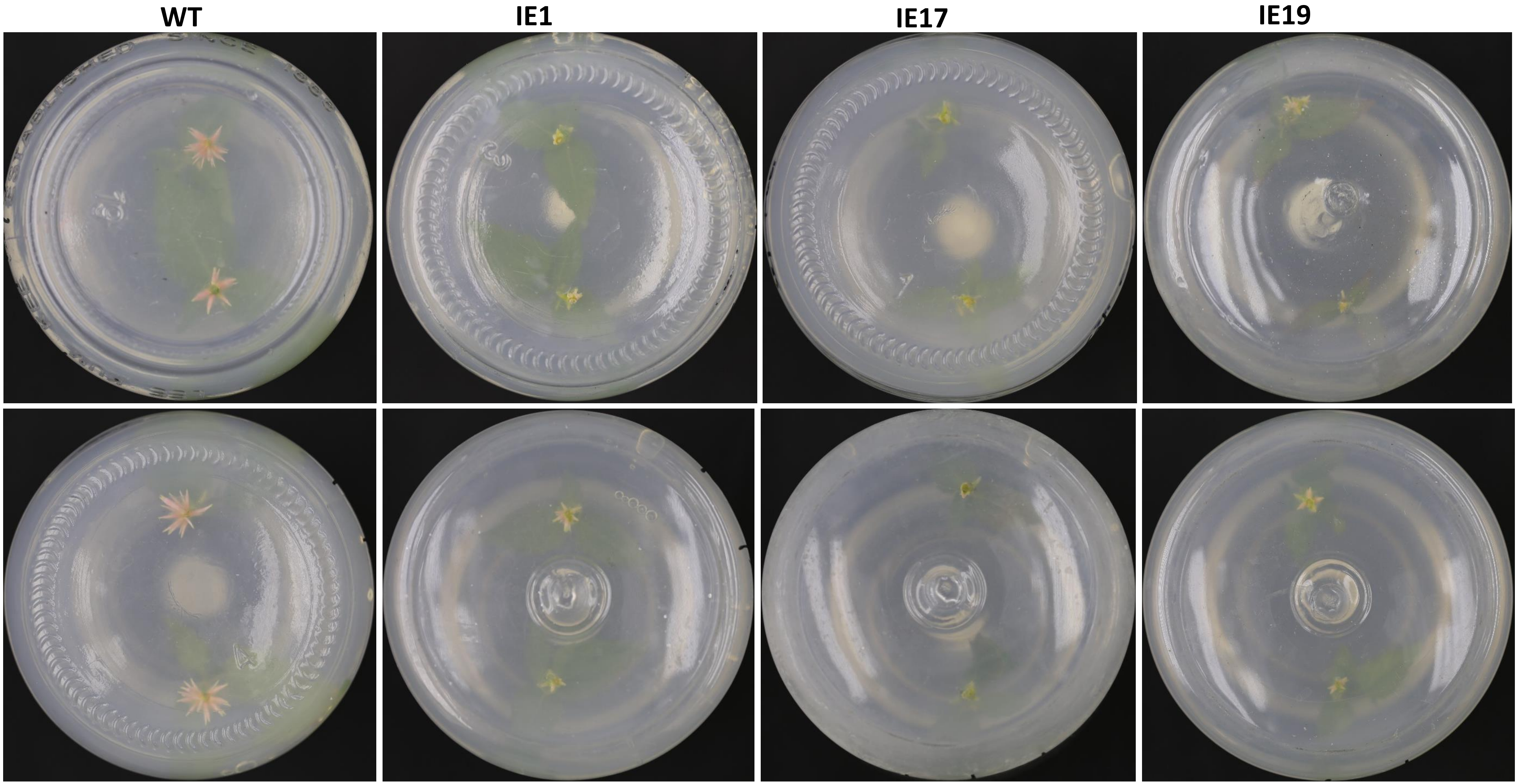

Figure S7. Adventitious root development in induced *bZIP53* overexpression transgenic lines in WPM
Cuttings of WT and 3 IE *bZIP53* lines were grown in WPM for 6 days. The cuttings initially showed the same growth status. The diameter of the tissue culture bottles was 7.0 cm.

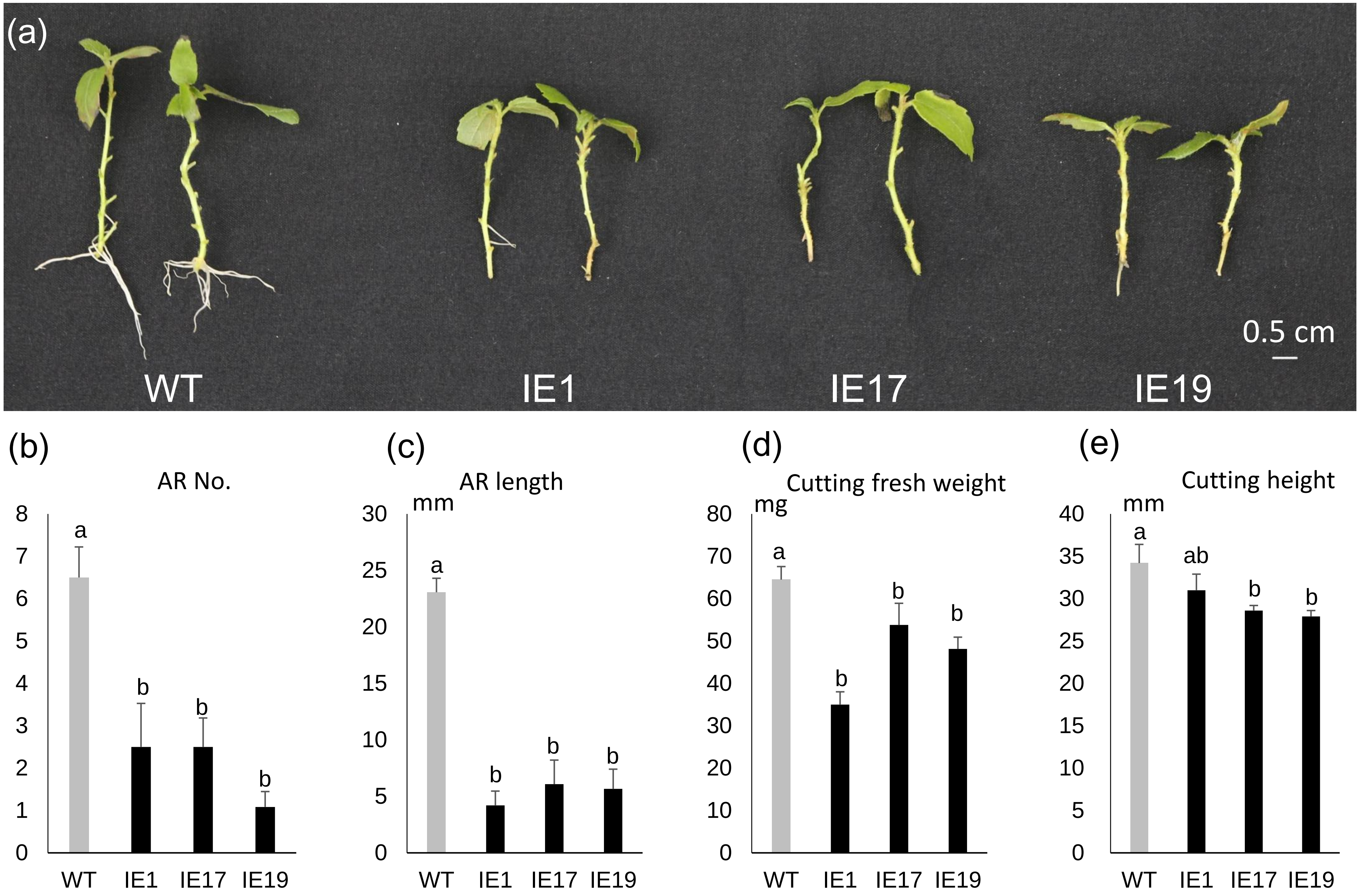

Figure S8. Phenotypic assay for poplar lines with induced overexpression of poplar *bZIP53* in soil (a)The AR phenotype for the representative plants of the 4 poplar lines, WT, IE1, IE17 and IE19. (b), (c), (d) and (e) represent the mean AR number, fresh weight, length and cutting length for the 4 lines propagated in soil for 12 days. The propagated plant materials initially showed the same growth status. The bars above the box indicate S.E. (standard error). All multiple comparisons were performed at the P=0.05 level.

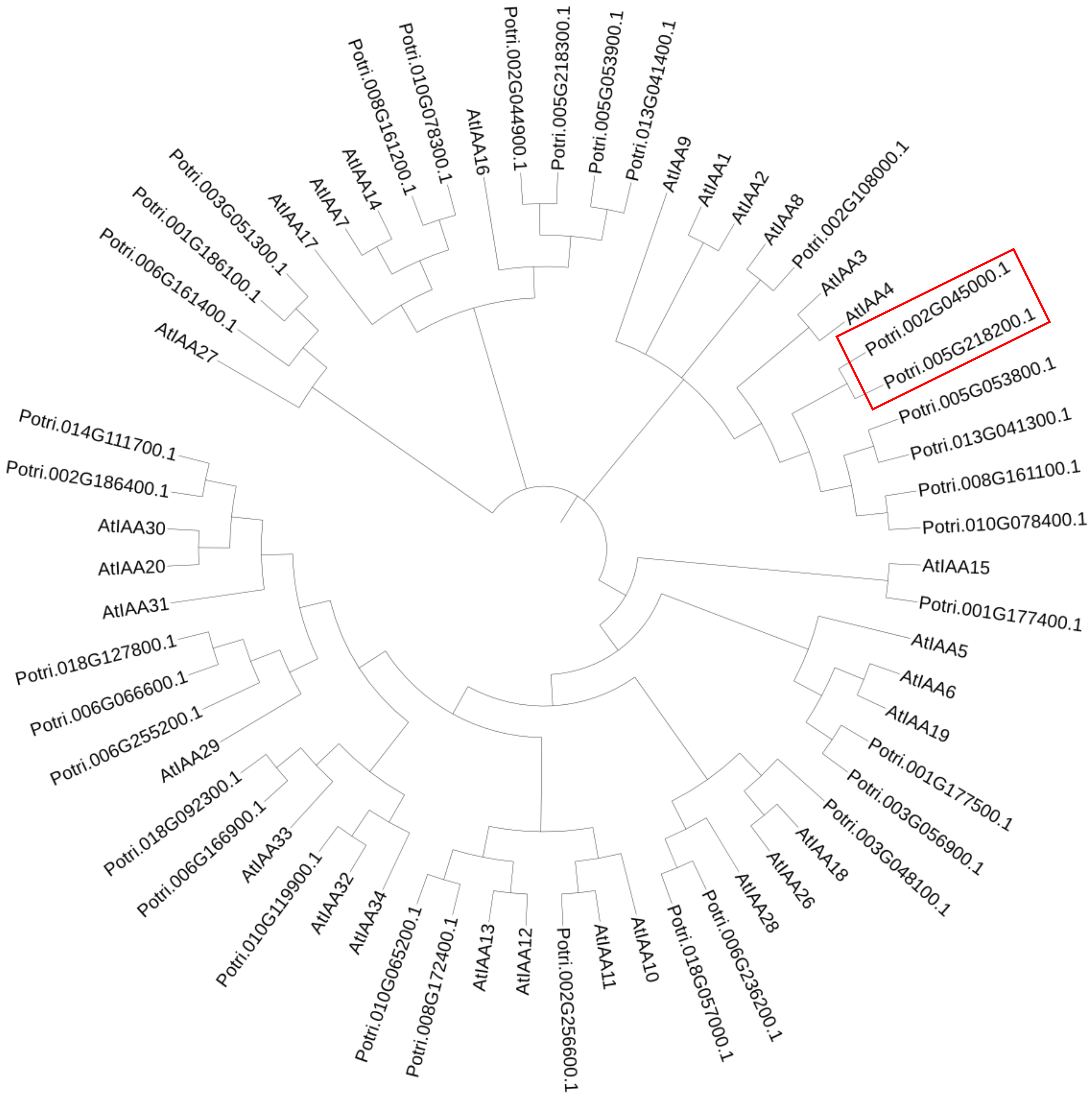

Figure S9. The phylogeny of Aux/IAA genes in *A. thaliana* and *P. trichocarpa*
Protein sequences were used to construct the phylogeny with the neighbour-joining method and 1000 bootstrap replications.

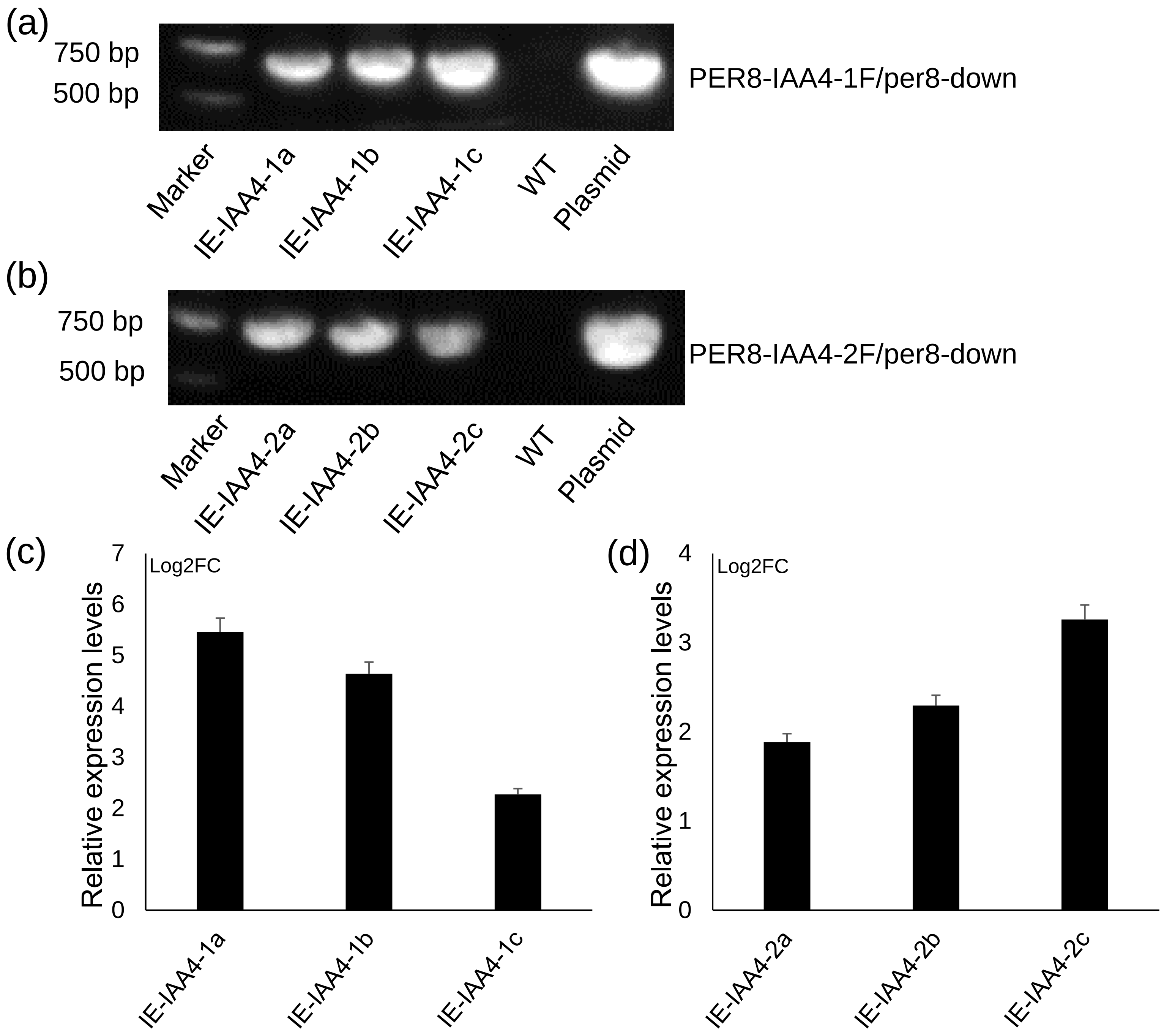

Figure S10. Positive transgenic screening of induced overexpression of *IAA4-1/2* transgenic lines in poplar 'Shanxinyang'

(a)and (b) PCR confirmed the 3 positive transgenic lines for *IAA4-1* and *IAA4-2* IE lines, respectively. (c) and (d) RT-qPCR assay for the expression of *IAA4-1* and *IAA4-2* IE lines, respectively. Other information is similar to Fig. S6.

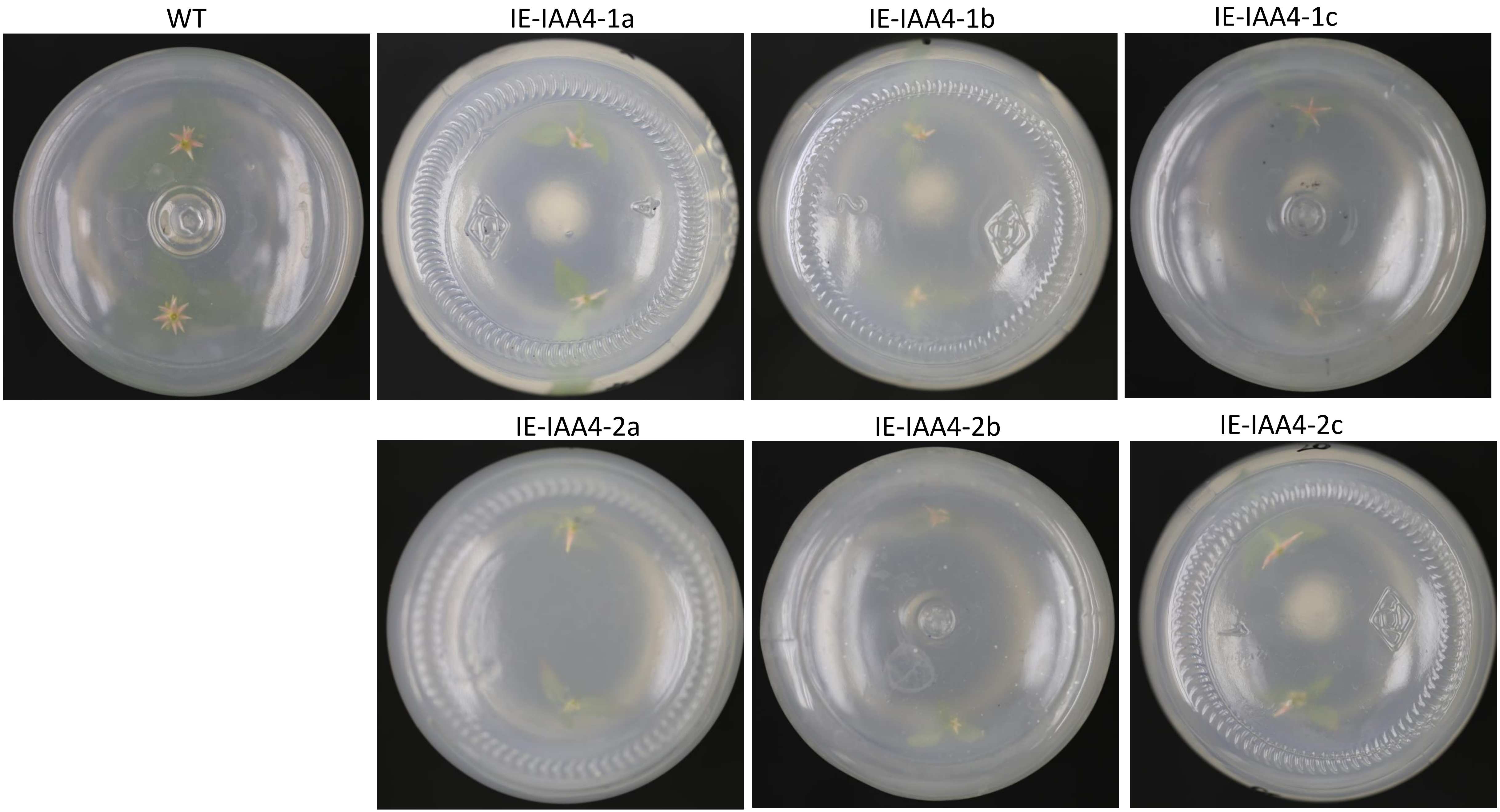

Figure S11. Adventitious root development in induced overexpression of *IAA4-1/2* transgenic lines in WPM
The details are as described for Fig. S3.

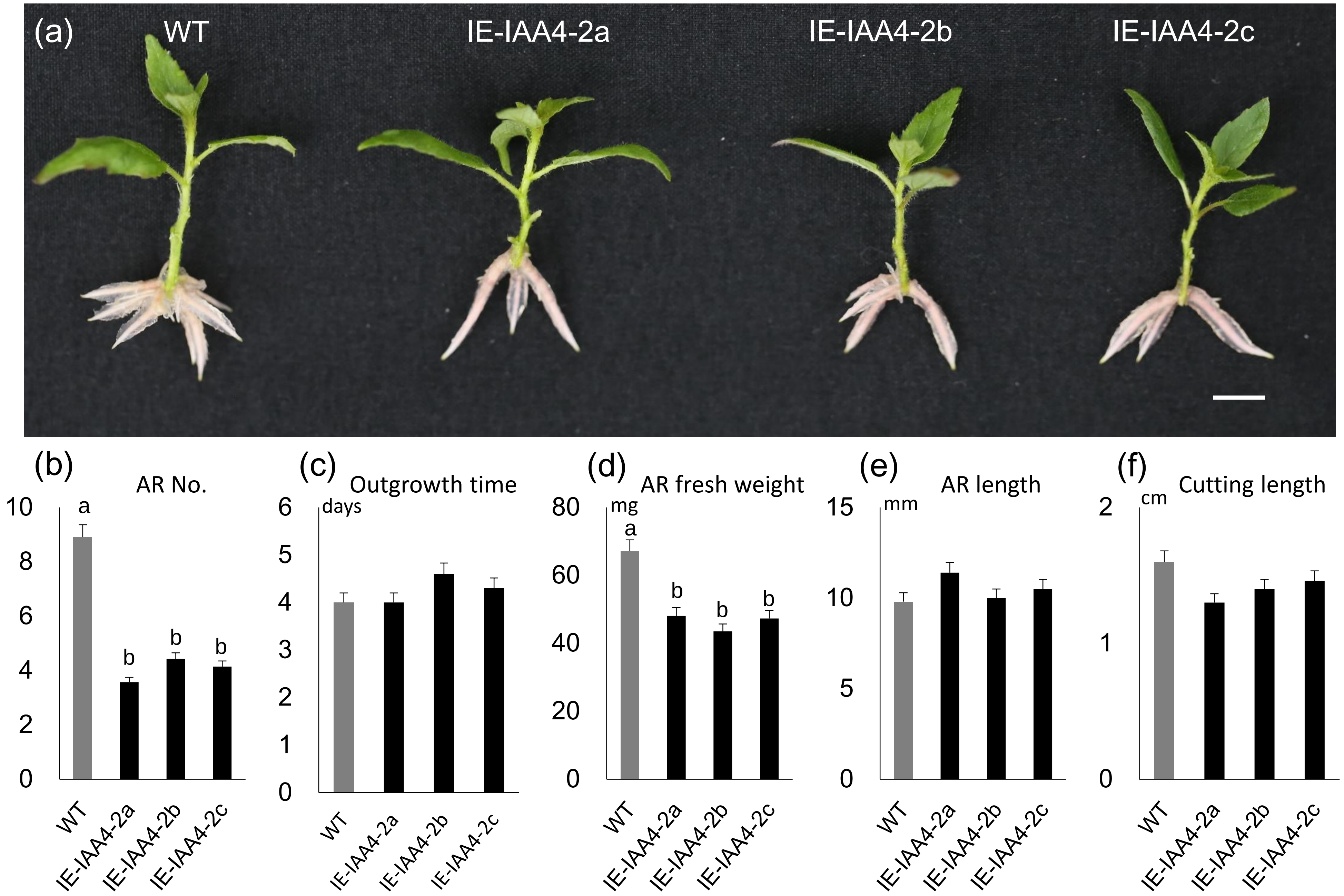

Figure S12. Phenotypic assay for poplar lines with induced overexpression of poplar *IAA4-2* in WPM The details are as described for Fig. 8.

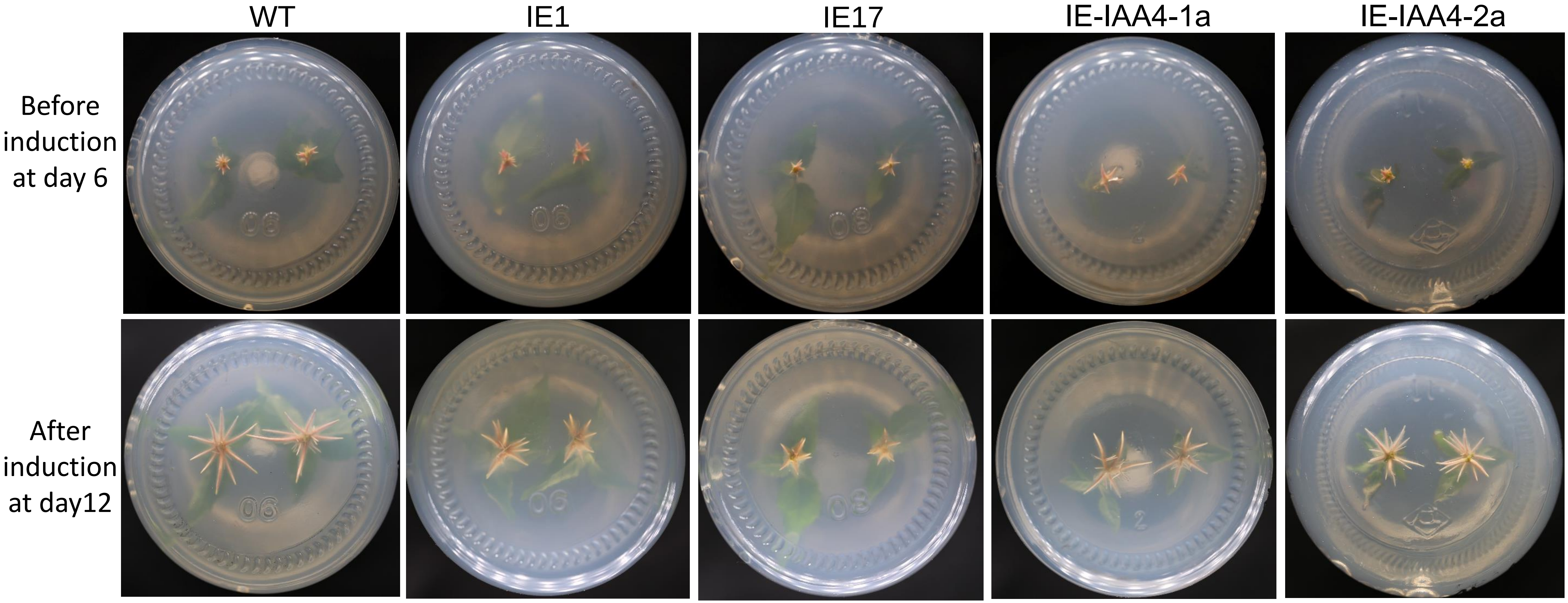

Figure S13. Adventitious root development for IE transgenic lines in WPM with phased oestradiol supplementation Cuttings of WT, IE1, IE2, IAA4-1a and IAA4-2a lines were propagated in WPM 6 days for induction of ARs (up) and then were transferred to WPM with 10 μM oestradiol for 6 days (down).

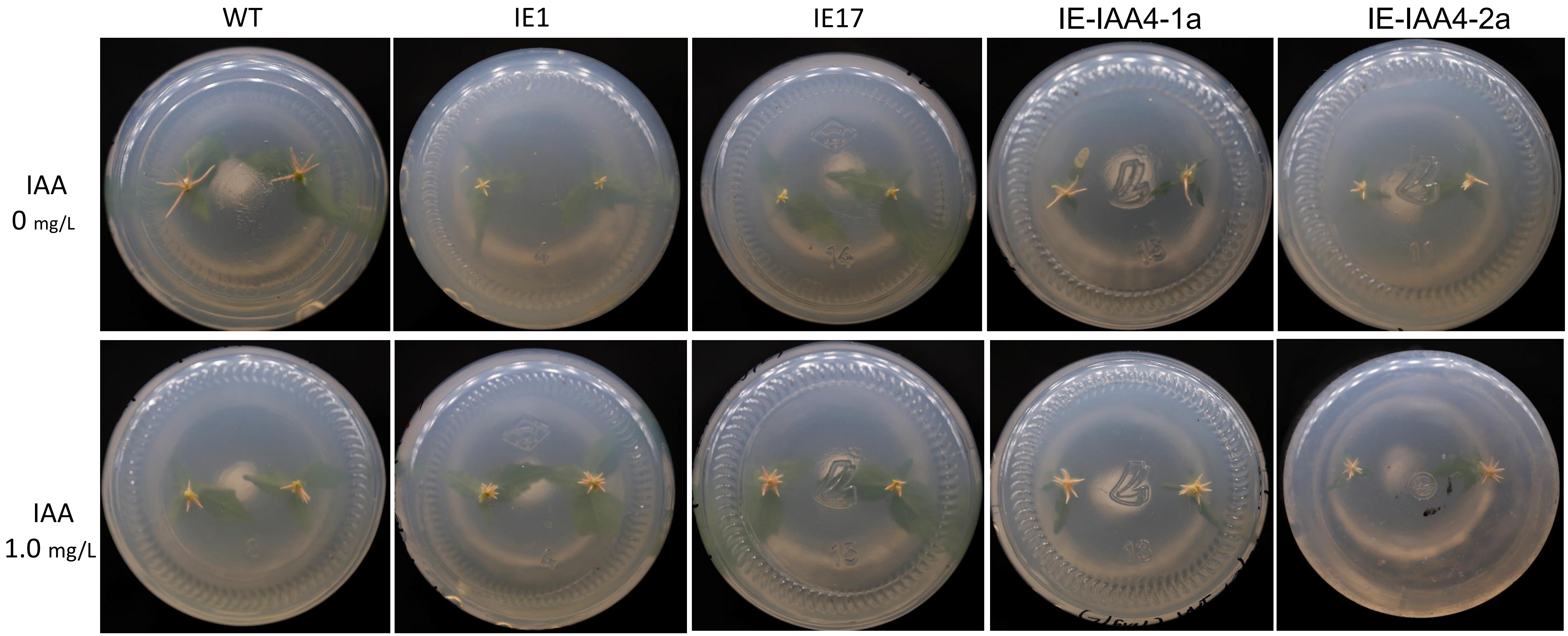

Figure S14. Adventitious root development for IE transgenic lines in WPM with or without supplementation of indole acetic acid (IAA)

Cuttings of WT, IE1, IE2, IAA4-1a and IAA4-2a lines were propagated in WPM with (down) or without (up) supplementation of IAA for 8 days.
