# Supplementary material for: The bZIP53–IAA4 module inhibits adventitious root development in Populus
Source: J Exp Bot. 2020 Feb 20;71(12):3485–98. doi: 10.1093/jxb/eraa096 (PMC7307859; doi:10.1093/jxb/eraa096)
Supplement: eraa096_suppl_Supplementary_Data_S1 [file eraa096_suppl_supplementary_data_s1.pdf]

> Poplar ’NL895’ *bZIP53* (*Potri.002G196200*) CDS
atgtctgcaaggcaagcggcgagctcaggatcggatagtgatccgcgatatgctaatgtcgatgagaggaaaaggaagaggatgatatcca
acagggaatctgcgaggcgttccaggatgaggaagcagaaacaattggagggtttggtcaatgaagtgagcaaattgcagaacgagaaca
atcagttgatgcagggtatcaatgttggccgacaacgcctcatggcgatggagtcagccaacaatgtcctgagggctcaggctgtggaattg
actgagaggctgcggtcgttgaactctgtgttgcagattgtggaggatgttagtggtctctctatggagatacccgaaatacccgatcctttgtt
gaagccgtggcagctcccctgttcagtaatgcccattatggcatctgctgatatgtttcaatattag

> Poplar ’NL895’ *IAA4-1* (*Potri.005G218200*) CDS
atggagaggtcaatggcatacctaagcgatctaaatctaaaggcaacagaactgaggttagggatgcctggaagtgatgagccagagaaa
ccatcaactactcctagtgttaggagtaataagagagcctcgccagaaatatcagaggagtccaggtccaagggcagttatagtgtgtcctcc
aatgtcgaaaatggtgaaagagacattgcccccctgccaaggcacaagtagtgggatggccaccaatccgatcttataggaaaaattgctt
gcaacaaaagaaaaatgatcaagtggatggtgctggcatgtacgtaaaagtgagcgtggatggagctccttatctcaggaagattgatctta
aggtgtacaagagctacccagagctcctcaaagccttggagaatatgttcaagcttaccattggtgaatactcagagaatgaagggtacaat
ggatctgaatttgctcctacttacgaagataaagatggagactggatgctagttggcgacgttccatggaacatgtttatctcctcctgcaaga
ggctgagaattatgaaaggatcagaagctagaggattgggctgttga

>Poplar ’NL895’ *IAA4-2* (*Potri.002G045000*) CDS
atggagaggtcaatggcatacgaaagacatctaaatctaaaggcaacagaactgagattagggttgcctggaagcgatgagccagagaaa
ccatcaactactcctagtgttaggagtaacaagagagcatcaccagaaatatcagaggagtctaggtccaagggcagctctagtctgtcctc
caatgttgaaaattgtgaaggagatggtgcccctcctgccaaggcacaagtagtgggatggccaccaatccgatcttacaggaaaaattgct
tgcaaccaaagaaaaatgatcgagttgatggtgctggaatgtacgtcaaagtaagcgtggatggagctccttatctcaggaagattgatctt
aaggtgtacaggagctacccagagctcctcaaagcattggaagatatgttcaagcttaccatcggagagtactcagagaaggaaggataca
atggatctgactttgctcctacttacgaagataaagatggagattggatgcttgttggagacgttccatgggacatgtttatctccacctgcaag
aggctgagaattatgaaggggtcagaagccagaggattgggctgttga

>Poplar ‘NL895’ *IAA4-1* promoter sequence
cttgctaccaaaatacttaatttcaaacctgccaagacagcagaggctgtatgtgtttacacctgtgaagctctaagtatcatggccaaaaca
aaaaattgtggatgagaattacaaaagtaacaatcagatagcactatcgcatttttatcatacagagaagcagcaggttgaatttgttataca
ttcctatcgttcaattaattgcaaaaattgagaagattttgtgaaccatctggggctactggctggctaggcttgtgaattatgcaccacaagat
ttagccaacaaaagatcttgccttgagtagagtttggtcgtggggcttccaaatatgcaggcatttatgtgtactccatgcctgactagctcgg
agatggattcgaagttaaaaaggcggcatctttccttgtaataaatgtgtggcaggcacatcctgatgttgaattccgtttacctacattgcctt
ggagtagagagtagaagccaaattctattttaacaatgcctttacaaagaacaagagattggcatttatttttgtacattgaacgccaaaattc
tgaagaaagaaatgatataatttcaagttggaacatgaaccctaagcgttgaataattaatgatacacagattcacacgaggatgattcattc
caaagtcatgttaccgttcgacaaatcctcttgtgaatttgtcaaatgtcaatggaggcatgcatgctacaattttagatcaaggtttaactcgt
agaaaattctgcaaatgcacaattcattgcactttttgactcgaattcctcttgtaggtcatttaattttttttattaattgattgtcaactaacagta
aacagatttattcatggtgaagtaagttgattaaaggtagacaatcttaaaaaaaaaaggaaaaaaaaaaataatgtatgactcagcaaggc
aagagctgaccatgccacatgtcatgtaccgtccacaagaccagatcaagttcgaaaaaattagaggtcccacatt**cacgtg**ggaccctcag
ttcacatggggatgttcccatcaagccctcaagagccaacgttccttgatttgcctctccaataaccaaatctcagccgttcagtcctggtcccg
tgtcttcaataccatcggctgtgcttgtctcttctttgtccccctcgcgagcagcaccaaactccaatgtgcttctcatttgacaagcagccatat
ctgttgctgactttcacttaaatgattcagctccacgcgcacagctgaagcgtgttctgtgagccccacctggaaaccccacttggaagtata
tgattggtttagaaggtagagcgtgtggctcaggtttgtggggttatcatttctgatcgatgaagttcatgcacgaagagaacgagtcacctgg
atattgtcccttgttaatcaccccctataaaactattcgtacatcccatctttctcatcaacacgccaaccgtttcatttcatttcaagaattaatta
gttctttcaactctaagcagaaagtgtcgagaagggaaaaacacagagaaatagagattatctggtttgcatcaagttagtgttcaagaatat
ttgtgataagc

Note: The G-Box motif was in bold and underlined. The 5'UTR was in yellow background and this UTR was annotated according to the corresponding region in *P. trichocarpa* genome version 3.0. Below is the same.

>Poplar 'NL895' *IAA4-2* promoter sequence
cgcaagacttgactaaacaaagattttgccttgagtaggtggtcatggggctcccatggatgcaggcatttactcttggctaggagatagact
caaagttaaatggttgcatctttccctgtaatt**aacgtg**gggcagtgttgttggaaggaggaacatcttgatgttgaattctacttgcctacat
tgcctttcagcagaaacccttttttttttttaacaattaacaaaaaccaaaagtatggtgaccctgctgtttaacacttcacaaaatattatttat
tataataatatatttatttttaatttttatcatttgatttattaaaaattaggttttataatttgttgtattttattttttatatggttatatcagttttatt
attcacattgttagtcttgtaaattaacccgatttgattttaaattttatttaaaataatattttttttaattttatcatttaatattaaattaattaaa
aattaaaatttatgatttgtttcattttatttttttatacattgagagctttgttaagctcaacaaggcaagagctgaccatgccacatgacattac
acatgtcatgtaccgtccacgagaccaaatcaaattccaaacacagagggtcccatttatgtcaaacccatatgaccatgcattgccaatcaa
aggcatccacgagggaccctcaggtcacatggggatgtccccatcaaaccctcatgatccaacgttccttgatttgcctctcaaagaatcaaa
tctcatccgttcgttcctggtccccagccttcaataccatcggccgtgtttgtctcttctttgtccccctcactgacaacaccaaactccattgtgc
gtctcgtttgacaagcaatgatatctgttgccgtctttcacttcaatgattcagctccacgcgcataactcaggcgagtttctgtgagcccaacct
gcaaaccctacttggcaatatgtgattggaccagagggtagagcgtgtggcacaggttcgtggggatatcattcctgatcaatgaaattcatg
cacgaacagaataagtcacctggatattgtcccttgttaacaacccttataaaactatttctgcatcgcctctttctcagcatcacaacaacagt
ttcaagaaatagttcaactttgattgatttggaatattcaagaattccctctgcgcaagaggttgtagccaaaagagagattaattattgtttgt
agccaaaagagagattaattattgtttgtagccaaaagagagattaattattgtttggcatcatcaagttttgctctaagatttgtgataagca
Note: legend is similar to *IAA4-1* promoter sequence

>G-BoxS sequence for Y1H
taatgtatgactcagcaaggcaagagctgaccatgccacatgtcatgtaccgtccacaagaccagatcaagttcgaaaaaattagaggtccc
acatt**cacgtg**ggaccctcagttcacatggggatgttcccatcaagccctcaagagccaacgttccttgatttgcctctccaataaccaaatct
cagccgttcagtcctggtcccgtgtcttcaataccatcggctgtgcttgtctcttctttgtccccctcgcgagcagcaccaaactccaatgtgctt
ctcatttgacaagcagccatatctgttgctgactttcacttaaatgattcagctccac
Note: The core bases in G-Box motif were in bold and underlined

>mG-BoxS sequence for Y1H
taatgtatgactcagcaaggcaagagctgaccatgccacatgtcatgtaccgtccacaagaccagatcaagttcgaaaaaattagaggtccc
acattc**aaaa**gggaccctcagttcacatggggatgttcccatcaagccctcaagagcca**aaaa**tccttgatttgcctctccaataaccaaatc
tcagccgttcagtcctggtcccgtgtcttcaataccatcggctgtgcttgtctcttctttgtccccctcgcgagcagcaccaaactccaatgtgctt
ctcatttgacaagcagccatatctgttgctgactttcacttaaatgattcagctccac
Note: The mutated core bases in G-Box motif were in bold and underlined
